# Gut microbiota in HIV–pneumonia patients is related to peripheral CD4 counts, lung microbiota, and in vitro macrophage dysfunction

**DOI:** 10.1186/s40168-019-0651-4

**Published:** 2019-03-11

**Authors:** Meera K. Shenoy, Douglas W. Fadrosh, Din L. Lin, William Worodria, Patrick Byanyima, Emmanuel Musisi, Sylvia Kaswabuli, Josephine Zawedde, Ingvar Sanyu, Emily Chang, Serena Fong, Kathryn McCauley, J. Lucian Davis, Laurence Huang, Susan V. Lynch

**Affiliations:** 1 Division of Gastroenterology, Department of Medicine, University of California San Francisco (UCSF), San Francisco, CA 94143 USA; 2 Biomedical Sciences Graduate Program, UCSF, San Francisco, CA USA; 3 Infectious Diseases Research Collaboration, Mulago Hospital, Makerere University, Kampala, Uganda; 4 HIV, Infectious Diseases and Global Medicine Division, Department of Medicine, San Francisco General Hospital, UCSF, San Francisco, CA USA; 5 Division of Pulmonary and Critical Care Medicine, Department of Medicine, San Francisco General Hospital, UCSF, San Francisco, CA USA; 6 Department of Pulmonary, Critical Care and Sleep Medicine, Yale School of Medicine, New Haven, CT USA; 7 Current address: Fred Hutchinson Cancer Research Center, Seattle, WA USA

## Abstract

Pneumonia is common and frequently fatal in HIV-infected patients, due to rampant, systemic inflammation and failure to control microbial infection. While airway microbiota composition is related to local inflammatory response, gut microbiota has been shown to correlate with the degree of peripheral immune activation (IL6 and IP10 expression) in HIV-infected patients. We thus hypothesized that both airway and gut microbiota are perturbed in HIV-infected pneumonia patients, that the gut microbiota is related to peripheral CD4+ cell counts, and that its associated products differentially program immune cell populations necessary for controlling microbial infection in CD4-high and CD4-low patients. To assess these relationships, paired bronchoalveolar lavage and stool microbiota (bacterial and fungal) from a large cohort of Ugandan, HIV-infected patients with pneumonia were examined, and in vitro tests of the effect of gut microbiome products on macrophage effector phenotypes performed. While lower airway microbiota stratified into three compositionally distinct microbiota as previously described, these were not related to peripheral CD4 cell count. In contrast, variation in gut microbiota composition significantly related to CD4 cell count, lung microbiota composition, and patient mortality. Compared with patients with high CD4+ cell counts, those with low counts possessed more compositionally similar airway and gut microbiota, evidence of microbial translocation, and their associated gut microbiome products reduced macrophage activation and IL-10 expression and increased IL-1β expression in vitro. These findings suggest that the gut microbiome is related to CD4 status and plays a key role in modulating macrophage function, critical to microbial control in HIV-infected patients with pneumonia.

**Electronic supplementary material:**

The online version of this article (10.1186/s40168-019-0651-4) contains supplementary material, which is available to authorized users.

## Summary

Gut microbiota composition of HIV-infected patients with bacterial pneumonia is associated with peripheral CD4 counts, airway microbiota composition, and in vitro macrophage dysfunction.

## Introduction

Our previous study of a large cohort of Ugandan HIV-infected acute pneumonia patients identified three compositionally distinct lower airway microbiota, each characteristically dominated by a different dominant bacterial family (*Prevotellaceae*, *Streptococcaceae*, or *Pseudomonadaceae*) that co-associated with a distinct group of bacteria [1]. Each of these three lower airway microbiota was associated with a distinct airway immune response, and patients with *Prevotellaceae*-dominated airway microbiota trended towards significantly higher patient mortality [1]. However, despite our large cohort, we did not identify a relationship between airway microbiota and CD4 cell status, leading us to speculate that systemic immune dysfunction underlying susceptibility to airway infection in HIV-infected patients is influenced by the gut microbiome and its associated products.

Emerging evidence in both animal [2, 3] and human [4, 5] studies has demonstrated a role for the gut microbiome in the development of airway immune dysfunction. More specifically, in neonates, perturbation to the gut microbiome is associated with a significantly increased risk of asthma development in childhood [4]. Such neonatal gut microbiome dysbiosis co-associates with metabolic dysfunction, including increased concentrations of a specific lipid, 12,13 DiHOME, which has been shown to reduce the frequency of regulatory T cell populations ex vivo in a dose-dependent manner [4]. In mice, manipulation of the gut microbiome either via diet or bacterial supplementation leads to enrichment of metabolites, including microbial-derived fatty acids that promote immune tolerance, protect against pathogen infection at the airway mucosal surface, and program pre-cursor cell populations in the hematopoietic compartment [2, 3]. Hence, emerging data implicates the gut microbiome and its metabolic products as playing a key role in influencing immune function at remote mucosal sites, including airway responses to pathogenic infection.

It is well established that HIV infection perturbs the gut microbiota [6], impairs mucosal barrier function, and increases microbial translocation [7]. Within patient populations in the USA, HIV-associated gut microbiota perturbation is consistently characterized by increased relative abundance of *Prevotella* and *Proteobacteria* and decreased *Bacteroides* abundance [6, 8, 9]. Vujkovic-Cvijin and colleagues demonstrated that gut dysbiosis is inconsistently and only partially rescued with antiretroviral treatment, while Dillon and colleagues revealed that gut microbiota dysbiosis associated with HIV infection is associated with chronic gut mucosal inflammation [10, 11]. Collectively, these independent microbiota studies demonstrate that HIV infection results in microbial dysbiosis within respiratory and gastrointestinal niches and co-associated immune dysfunction, but it has yet to be determined whether such large-scale perturbations occur in parallel and are related to HIV co-morbidities or to disease severity as defined by CD4+ cell counts.

We therefore hypothesized that lower airway microbiota dysbiosis observed in HIV-infected pneumonia patients is accompanied by a parallel gut microbiota perturbation. We further theorized that gut microbiota composition of HIV-infected pneumonia patients is related to peripheral CD4 cell count and that the products of the gut microbiome influence the behavior of immune cell populations crucial to control of microbial infection. To investigate this hypothesis, we collected paired lower airway and stool samples (*n* = 306) from a large cohort of Ugandan, HIV-infected patients (*n* = 153) with pneumonia, to address these hypotheses.

## Results

### Microbiota niche specificity is largely maintained in HIV-infected patients

**Fig. 1:**
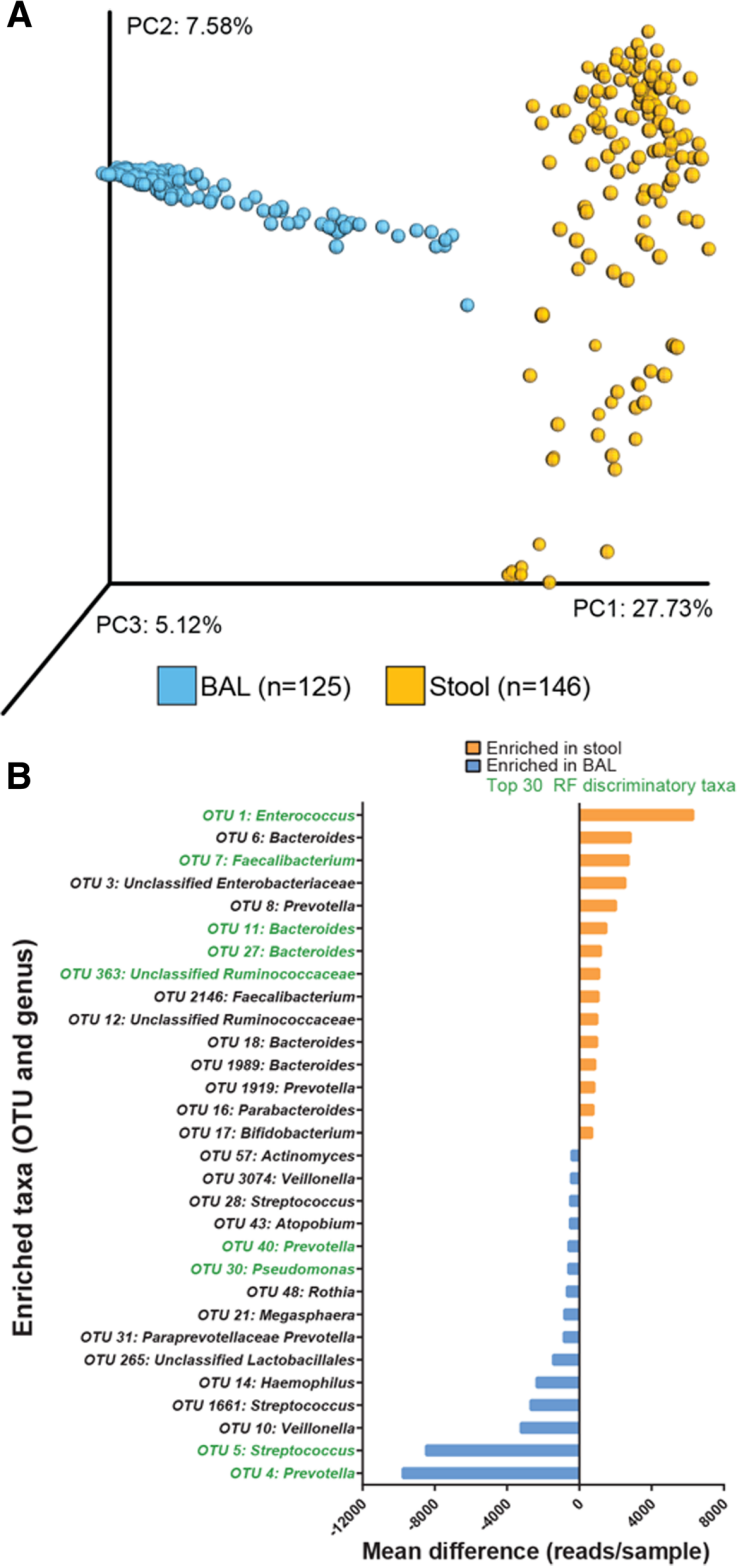
HIV-infected pneumonia patient stool and BAL exhibit body habitat specificity. **a** Principal coordinates analysis (PCoA) using Bray–Curtis dissimilarity of *n* = 271 bronchoalveolar lavage (BAL; *n* = 125) and stool (*n* = 146) bacterial community profiles (including 120 paired BAL–stool samples) representatively rarefied to 51,997 reads/sample. **b** Mean difference in reads/sample is plotted for the top 30 taxa differentiating BAL and stool bacterial communities, identified using linear mixed effects model and false discovery rate *q* < 0.05, read difference ≥ 100, and presence in ≥ 50% of the enriched group. PC, principal coordinate; taxa in green were also within the top 30 random forest predictive taxa differentiating BAL and stool samples

Bronchoalveolar lavage (BAL) and stool samples were obtained from Ugandan, HIV-infected patients admitted to Mulago Hospital for acute pneumonia (*n* = 153 patients). Study subjects possessed a median circulating CD4 count of 131 cells μl^−1^, and almost all were administered antibiotics prior to undergoing bronchoscopy (for patient characteristics see Additional file 1: Table S1). Bacterial microbiota profiles were generated using V4 16S rRNA amplicon sequencing for lower airway and stool samples. Combined BAL and stool sample operational taxonomic unit (OTU) picking (representatively rarefied to 51,997 reads per sample) and principal coordinates analysis [PCoA (Bray–Curtis dissimilarity)] of 16S rRNA bacterial sequences revealed that despite the severely immunocompromised status of this population, the bacterial communities in these body habitats are compositionally distinct (Fig. 1a). BAL and stool comparisons based on rarified 16S rRNA data was confirmed with DESeq2, which includes all sequence data and normalizes for differences in sequence depth (Additional file 1: Figure S1). OTU distribution modeling, as previously described [12], with validation by random forest analysis was used to identify those bacterial taxa that differentiated stool and BAL communities; taxa most enriched within the stool were traditional gut-associated microbes including *Bacteroides*, *Faecalibacterium*, and *Ruminococcaceae*, while BAL was characteristically enriched for airway-associated microbes including *Streptococcus*, *Veillonella*, and *Haemophilus* (Fig. 1b). These data demonstrate that despite severe CD4 suppression, this HIV-infected pneumonia study population largely retains site-specific microbiota in the lower airways and gastrointestinal tract.

### Lower airway microbiota stratify into distinct bacterial microbiota structures

**Fig. 2:**
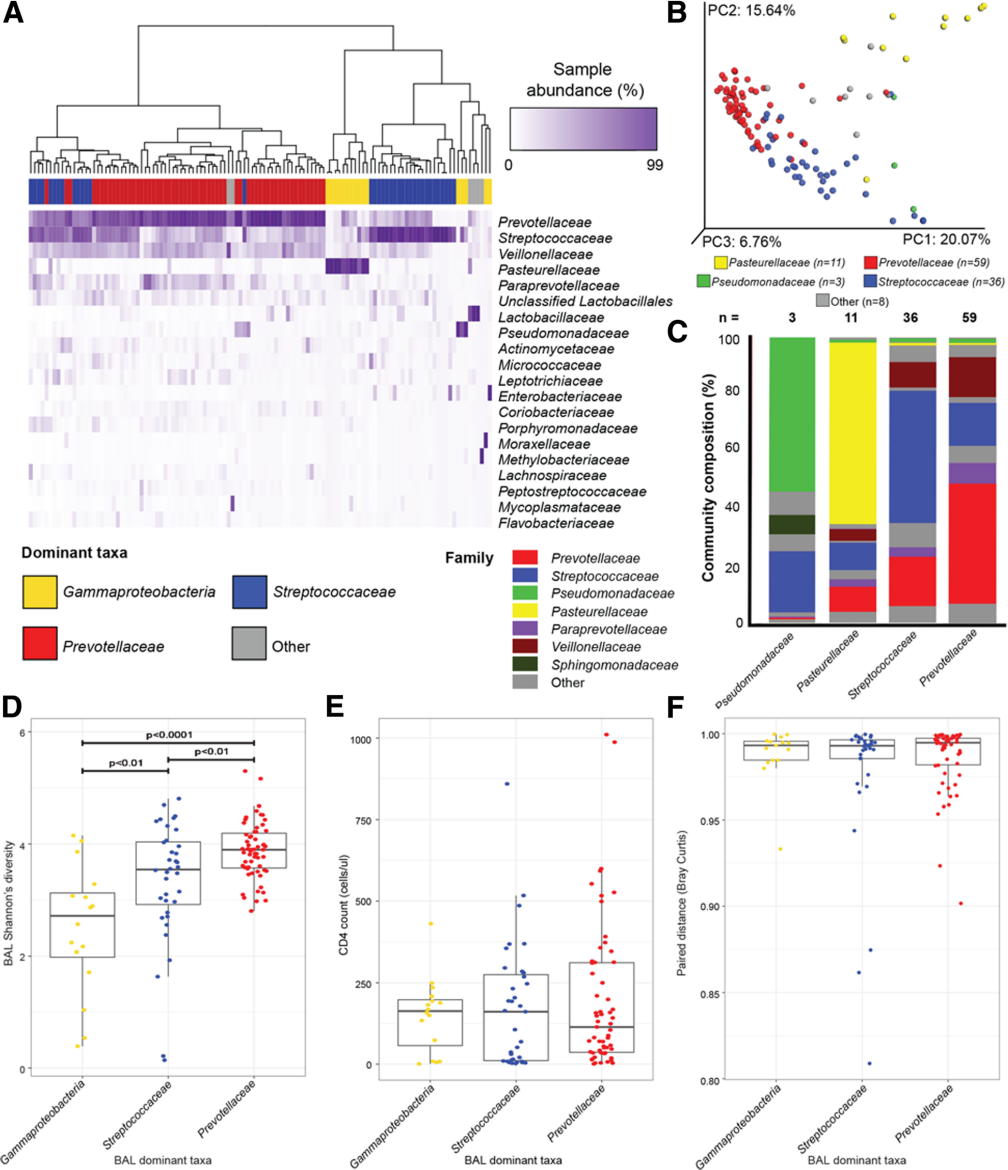
Three distinct airway microbiota exist in HIV-infected pneumonia patients and relate to microbiological factors, but not to peripheral CD4 count or gut microbiota composition. **a** Unsupervised hierarchical clustering (using Bray–Curtis dissimilarity (BC) and Ward 2 clustering) of BAL microbiota and abundance heat map of the top 20 bacterial families present in at least one sample at ≥ 3% relative abundance (ordered from highest to lowest abundance) indicate distinct but repeating patterns of microbial co-colonization in the lower airways of HIV-infected pneumonia patients. **b** Principal coordinates analysis (PCoA) of weighted UniFrac distance for *n* = 117 BAL samples confirms that significantly distinct airway microbiota co-associate with dominant bacterial family and explain a large proportion of taxonomic variation in lower airway bacterial communities (PERMANOVA, *R*^2^ = 0.658, *p* < 0.001). **c** Mean community composition at the family level for each of the distinct microbiota community states. Bacterial community states differ in **d** Shannon’s diversity (KW, *p* < 0.0001; Mann–Whitney *U* test *p* values plotted), but do not relate to **e** peripheral CD4 count (cells μl^−1^; KW *p* = 0.85) or to **f** similarity to paired stool sample (paired distance measured using Bray–Curtis; KW, *p* = 0.62). PC, principal coordinate; PERMANOVA, permutational multivariate ANOVA; KW, Kruskal–Wallis rank sum test

To investigate lower airway microbiota in greater depth, BAL 16S rRNA sequences were clustered into OTUs (independently of gut samples) and representatively rarefied to 67,135 reads per sample for a final high-quality data set of 117 airway samples. Consistent with our previous observations, the lower airway bacterial microbiota within this cohort displayed distinct compositional patterns related to the dominant bacterial family detected [permutational multivariate analysis (PERMANOVA, weighted UniFrac distance, *R*^2^ = 0.658, *p* < 0.001; unweighted UniFRAC *R*^2^ = 0.118, *p* < 0.002], and evidence of either a reciprocal gradient of *Prevotellaceae-* to *Streptococcaceae*-dominance across the majority of samples (*n* = 95), or microbiota dominated by genera belonging to the *Gammaproteobacteria* (*n* = 16; Fig. 2a). As previously described, a large majority of lower airway microbiota were dominated by *Prevotellaceae* or *Streptococcaceae* which consistently exhibited co-colonization with *Veillonellaceae* and *Paraprevotellaceae* (Fig. 2b, c). The remaining *Gammaproteobacteria*-dominated samples were primarily dominated by *Pasteurellaceae* (*n* = 11) or *Pseudomonadaceae* (*n* = 3), exhibited greater compositional variability, and clustered distinctly from the majority of *Prevotellaceae-* and *Streptococcaceae*-dominated samples (Fig. 2b, c), corroborating our previously published observations in an independent cohort [1, 13].

We had also previously reported that *Prevotellaceae-* and *Streptococcaceae-*dominated lower airway microbiota were associated with higher bacterial diversity, administration of the first-line pneumonia antibiotic ceftriaxone, and increased detection of cultivable fungi. In our current study, *Prevotellaceae-* and *Streptococcaceae-*dominated samples also exhibited significantly higher Shannon’s bacterial diversity compared with the *Gammaproteobacteria*-dominated samples (Kruskal–Wallis, *p* < 0.0001; Fig. 2d). Additionally, the majority of airway microbiota were *Prevotellaceae-* or *Streptococcaceae-*dominated, which we attribute to the near-universal administration of ceftriaxone (91%) in this cohort (see Additional file 1: Table S2 for stratification of clinical factors by airway microbiota).

To address whether these airway bacterial microbiota states differentially co-associated with fungi, we also amplified the ribosomal internal transcribed spacer 2 (ITS2) region from the same DNA extracted for 16S rRNA profiling from BAL samples. Only 26 samples produced a high-quality fungal profile (representatively rarefied to 1044 reads per sample). The low number of fungal positive samples is consistent with the clinical observation that our cohort is a bacterial, rather than fungal pneumonia patient population. Fungal communities were typically dominated by *Candida* (*n* = 19/26; Additional file 1: Figure S2A), and unsurprisingly, the dominant fungal taxon explained a large portion of mycobiota compositional variance across these samples (Bray–Curtis PERMANOVA, *R*^2^ = 0.673, *p* < 0.001; Additional file 1: Figure S2B). CD4 count was also found to be related to airway fungal composition (Bray–Curtis PERMANOVA, *R*^2^ = 0.220, *p* < 0.01; Additional file 1: Figure S2C). However, this association was likely driven by *Pneumocystis jirovecii-*dominated samples (*n* = 3), all of which were detected in the most severely immunosuppressed patients with very low CD4 counts (cells μl^−1^ < 34). Of note, while most of our BAL samples produced robust 16S rRNA amplicons, *Pneumocystis-*dominated samples did not produce a bacterial signal. No association was observed between lower airway fungal and bacterial composition, which may be due to the relatively small number of fungal positive samples and the high frequency of co-trimoxazole administration for *Pneumocystis* pneumonia prophylaxis (83%). Nonetheless, consistent with our previous observations, fungal detection was more common in patients with *Streptococcaceae-* and *Prevotellaceae*-dominated communities, though due to the small fungal-positive sample size, this association was not significant (chi-square test, *p* > 0.05; Additional file 1: Figure S2D).

Finally, we assessed whether lower airway bacterial community states were related to systemic immune dysfunction as indicated by CD4 status. Consistent with our previous study, circulating CD4 count (cells μl^−1^) was neither associated with airway microbiota composition variability nor with community state (PERMANOVA, *R*^2^ = 0.009, *p* = .21; Kruskal–Wallis, *p* = 0.85; Fig. 2e).

We next asked whether the three observed lower airway microbiota states were related to gut microbiota composition. As a measure of intra-personal airway–gut microbiota similarity, we calculated Bray–Curtis paired distance, which weights higher abundance, rather than phylogenetically related (UniFrac) or lower abundance (Canberra) taxa. Mean airway–gut microbiota paired distances did not significantly differ when patients were stratified by lower airway community state (Kruskal–Wallis, *p* = 0.62; Fig. 2f). Thus, our data indicates that variation in HIV–pneumonia associated lower airway bacterial community is neither related to CD4 count nor to the degree of similarity with gut microbiota composition. We therefore next determined whether gut microbiota related to disease severity and patient outcomes.

### Gut microbiota composition is related to clinical and CD4 status

**Fig. 3:**
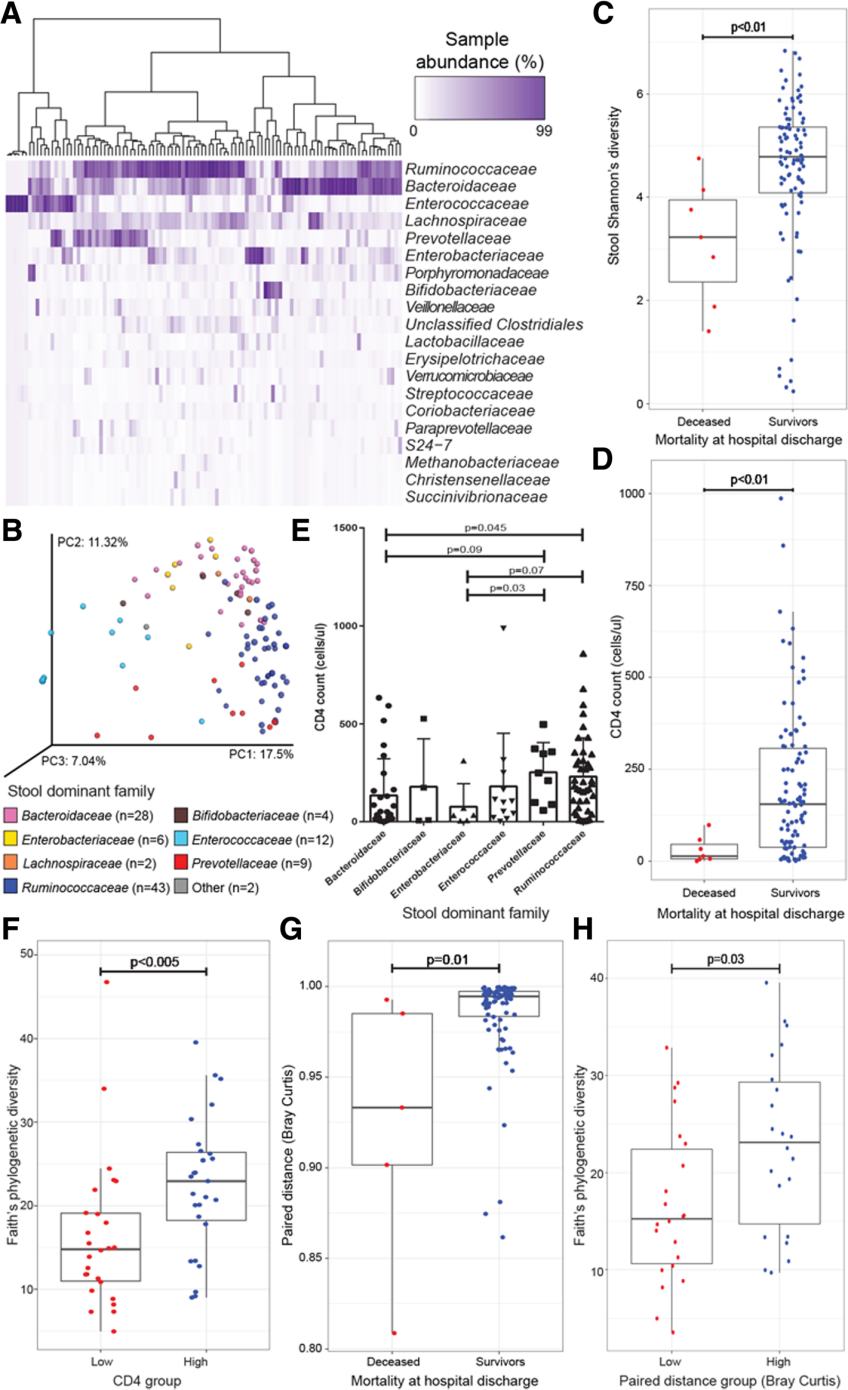
Stool microbiota composition is related to clinical and immunological factors and to CD4 status of HIV-infected pneumonia patients. **a** Unsupervised hierarchical clustering (using Bray–Curtis dissimilarity (BC) and Ward 2 clustering) and abundance heat map of the top 20 bacterial families present in at least one sample at ≥ 3% relative abundance (ordered from highest to lowest abundance) indicate patterns of microbial co-colonization in stool. **b** Principal coordinates analysis (PCoA) of BC dissimilarity for *n* = 106 stool samples illustrates that dominant bacterial family explains a high degree of variation in bacterial microbiota composition [PERMANOVA, *R*^2^ = 0.319, *p* < 0.001; other = *Streptococcaceae* (covered by *Enterococcaceae* on left) and *Porphyromonadaceae*]. Mortality at hospital discharge is significantly associated with **c** stool Shannon’s diversity (Mann–Whitney *U* test, *p* < 0.01) and **d** CD4 count (cells μl^−1^; Mann–Whitney *U* test; *p* < 0.01). **e** CD4 count (cells μl^−1^) is significantly related to stool dominant bacterial family (Mann–Whitney *U* test; KW, *p* = 0.02; only families with > 2 samples are included in this analysis). **f** CD4 count (quartile 1 versus 4) is significantly related to stool Faith’s phylogenetic diversity. Similarity between lower airway and stool samples (Bray–Curtis paired distance) is significantly related to **g** mortality at hospital discharge (MW, *p* = 0.01) and **h** to stool Faith’s phylogenetic diversity (MW, *p* < 0.03) when grouped by quartiles and quartile 1 versus 4 are compared. PC, principal coordinate; PERMANOVA, permutational multivariate ANOVA; MW, Mann–Whitney *U* test

To investigate patient gut microbial communities in greater depth, stool bacterial 16S rRNA sequences were clustered at 97% sequence identity into OTUs (independent of airway samples) and rarefied to 120,665 reads per sample (*n* = 106). Stool bacterial community composition was generally characterized by high relative abundance of *Ruminococcaceae*, *Bacteroidaceae*, *Enterococcaceae*, *Enterobacteriaceae*, or *Prevotellaceae* with *Ruminococcaceae* (Fig. 3a). PERMANOVA analysis of stool samples revealed that variation in community composition was related to demographic, clinical, and microbiological factors, but not to sample processing control variables (Table 1). Of these related factors, dominant bacterial family explained the greatest proportion of compositional variance (Bray–Curtis PERMANOVA, *R*^2^ = 0.319, *p* < 0.001; Fig. 3b) and was related to the percent dominance of the prevailing bacterial family (Kruskal–Wallis, *p* < 0.001), suggesting that dominant bacterial families within the perturbed gut microbiota of HIV-infected patients relate to the overall structure of these bacterial communities.

**Table 1:** Clinical and microbiological features are significantly associated with variation in stool microbial composition in HIV-infected patients with bacterial pneumonia

Category	Factor	PERMANOVA^a^	
		*R* ^2^	*p* value
Clinical			
	CD4 count (grouped quartile 1 versus 4)	0.052	0.002
	Ceftriaxone at time of sampling	0.019	0.014
	Mortality at hospital discharge	0.018	0.017
	CD4 count (cells/μl)	0.017	0.025
	Pulmonary tuberculosis	0.009	0.499
	Ceftriaxone within 3 months prior to hospitalization	0.008	0.634
Microbiological			
	Dominant family	0.319	0.001
	Shannon diversity	0.145	0.001
	Paired BAL dominant family	0.122	0.033
	Total observed species^b^	0.111	0.001
	Faith’s phylogenetic diversity	0.104	0.001
	Percent dominance	0.072	0.001
	Bray–Curtis distance to paired BAL sample (grouped quartile 1 versus 4)^c^	0.054	0.002
	Bray–Curtis distance to paired BAL sample (continuous)	0.026	0.007
	Faith’s phylogenetic diversity of paired BAL sample^d^	0.021	0.028
	Fungal percent dominance	0.022	0.1
	Fungal dominant genus	0.095	0.284
Processing			
	Sequenced on NextSeq run 1	0.012	0.132
	Sequenced on NextSeq run 2	0.012	0.132
	ITS2 fungal amplicon sequenced	0.013	0.162
	16S amplification plate	0.022	0.221
	16S primer plate	0.022	0.221
	Extraction plate	0.007	0.824

^a^Permutational multivariate ANOVA results for Bray–Curtis distance shown; representative of weighted and unweighted UniFrac and Canberra distances

^b^Similar results for chao1 richness estimate

^c^Similar results for weighted and unweighted UniFrac and Canberra distances

^d^Stool composition is not significantly related to BAL richness or Shannon diversity

Stool fungal microbiota composition was also investigated using ITS2 rRNA sequencing. Compared with airway samples, a much larger proportion of gut samples produced a high-quality mycobiota profile (*n* = 90; rarefied to 2565 reads per sample). Stool fungal community composition was also related to dominant fungal family (Bray–Curtis PERMANOVA, *R*^2^ = 0.442, *p* < 0.001; Additional file 1: Figure S3A), and, similar to BAL fungal communities, was most frequently dominated by *Candida* (72/90 samples with 94% mean dominance; Additional file 1: Table S3). Unsurprisingly, given the almost universally monolithic presence of *Candida* in the gut mycobiota of these patients, stool fungal community composition was not related to patient factors, bacterial microbiota, or paired airway fungal community composition (data not shown). Contrary to the clear anatomical site specificity exhibited by bacterial microbiota, principal components analysis of mycobiota profiles indicated no such no site specificity (Additional file 1: Figure S3B and C). Since stool fungal communities were highly homogenous across anatomical niches and patients in our study and did not relate to clinical or immunological factors, we focused the remainder of our studies on bacterial microbiota.

To better understand patient disease, variation in stool bacterial composition and microbiological characteristics were examined in relation to patient treatment and outcomes. Administration of ceftriaxone was significantly related to stool microbiota composition though it only explained a minor proportion of variance (Bray–Curtis PERMANOVA, *R*^2^ = 0.019, *p* = 0.01; Additional file 1: Figure S4) and did not significantly lower bacterial diversity (Mann–Whitney; *p* > 0.05). These data indicate that antimicrobial administration to HIV-infected patients with pneumonia may not exert large effects on an already perturbed gut microbiome. Despite a low rate of mortality within this cohort (6%), mortality at time of hospital discharge was related to stool microbiota composition (Bray–Curtis PERMANOVA, *R*^2^ = 0.018, *p* = 0.017) and to lower Shannon’s diversity compared with survivors (Mann–Whitney, *p* < 0.01; Fig. 3c). While stool bacterial microbiota was related to patient mortality, the low number of deceased patients limited the power of this study to detect taxa that consistently differed between deceased and survivors. Instead, we examined relationships between the gut microbiota and circulating CD4 count as a marker of disease severity. As expected, deceased patients in our cohort had significantly lower CD4 counts (cells μl^−1^) compared to survivors (Mann–Whitney, *p* < 0.01; Fig. 3d). The cohort’s mean CD4 count was 186, median was 131, and range was 1–1010 cells μl^−1^ (Additional file 1: Figure S5A). Contrary to the airway microbiota, CD4 count was significantly associated with variation in gut microbiota bacterial composition (Bray–Curtis PERMANOVA, *R*^2^ = 0.017, *p* = 0.025; Additional file 1: Figure S5B) and with dominant bacterial family, with the lowest CD4 counts associated with *Enterobacteriaceae*-dominated gut microbiota (Kruskal–Wallis, *p* = 0.02; Fig. 3e). Since the CD4 distribution within the study population was severely skewed by high CD4 counts, patients were also sub-grouped by quartile into CD4 low (cells μl^−1^ < 34, quartile 1), CD4 intermediate (34 < cells μl^−1^ < 293, quartiles 2 and 3), and CD4 high (cells μl^−1^ > 293, quartile 4) to better understand the extremes of patient disease within this population and identify relationships with gut microbiota based on CD4 count. As with continuous CD4 count, these CD4 subgroups were also significantly associated with stool bacterial composition (Bray–Curtis PERMANOVA, *R*^2^ = 0.052, *p* = 0.002; Additional file 1: Figure S5C) and the increase in *R*^2^ (3.5%) gained by grouping CD4 count illustrates that consistent compositional patterns are associated with low versus high CD4 counts. Patients with high CD4 counts possessed significantly higher gut microbiota diversity than those with low CD4 counts (Mann–Whitney, *p* < 0.005; Fig. 3f), confirming that stool bacterial microbiota diversity and composition are related to peripheral CD4 count and disease severity.

### Gut microbiota composition relates to airway bacterial community composition

Since stool bacterial microbiota has been shown to regulate mucosal immunity at the airway surface [2, 3] and was related to patient outcomes and to CD4 status in this study, we next asked whether it related to airway microbiota composition. To investigate this, the inter-personal gut–airway Bray–Curtis similarity indices previously calculated were again used for *n* = 120 patients who provided paired airway and stool samples for analyses. The distribution of these paired distances (P-D) was skewed towards lower distances (indicative of greater compositional similarity across paired gut and airway microbiota), with mean distance 0.981, median 0.994, and range 0.810–0.999 (Additional file 1: Figure S6A). Variation in stool microbiota composition was significantly related to P-D (Bray–Curtis PERMANOVA, *R*^2^ = 0.026, *p* = 0.007; Additional file 1: Figure S6B), indicating that increased airway–gut microbiota compositional similarity (a proxy for shared bacterial taxonomy across gut and airway body habitats) is related to gut microbiota composition. P-D was also related to mortality at hospital discharge; deceased patients exhibited lower P-D values, i.e., greater microbiota compositional similarity across gut and airway habitats (Mann–Whitney, *p* = 0.01; Fig. 3g), though with the low number of deceased at hospital discharge within this cohort, this finding is difficult to interpret. To better understand the relationship between paired distance and stool composition, patients were stratified based on paired distance quartiles into low [paired distance (P-D) < 0.9816, quartile 1], intermediate (0.9815 < P-D < 0.9972, quartiles 2 and 3), and high (P-D > 0.9971, quartile 4) strata. P-D sub-groups were related to stool bacterial composition (PERMANVOA; Bray–Curtis; *R*^2^ = 0.054, *p* = 0.002; Additional file 1: Figure S5C), and patients with low P-D also possessed significantly lower gut microbiota diversity than those with high P-D (Mann–Whitney, *p* = 0.03, Fig. 3h). Interestingly, stool and BAL dominant families alone were not sufficient to explain these relationships between gut and airway microbiota (Additional file 1: Figure S7A and B), indicating that lower abundance microbes within these communities are driving community similarities. These data indicate that the composition of the gut microbiome of HIV-infected pneumonia patients is related to airway microbiota composition, to the degree of shared taxonomy across these body habitats, and to mortality outcomes in our patient cohort.

### Low CD4 count is associated with increased gut–airway microbiota similarity and taxon sharing across body sites

**Fig. 4:**
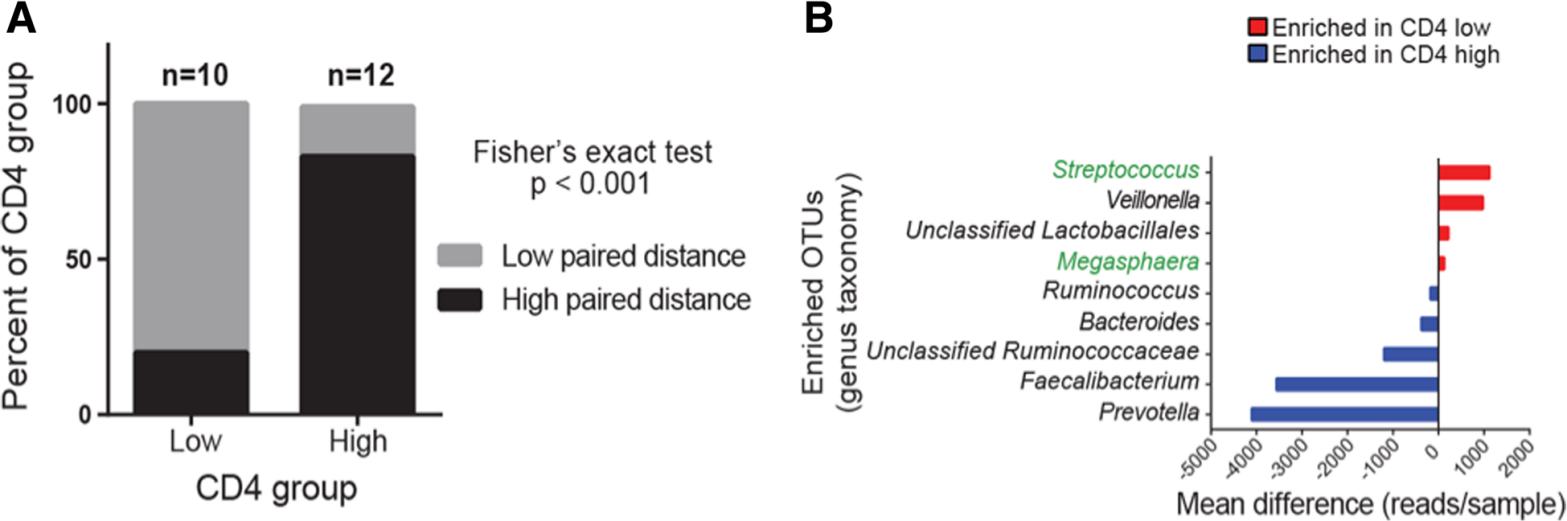
Patients with low CD4 counts exhibit a greater degree of gut–airway microbiota similarity, loss of gut microbiota commensals, and evidence of *Streptococcus* and *Veillonella* enrichment in both gut and airway microbiota. **a** Patients with low CD4 counts are significantly more likely to have low airway–gut microbiota paired distance (quartile 1 versus 4 for each factor, Fisher’s exact test, *p* < 0.001). **b** CD4 low (versus high) patient stool bacterial communities exhibit enrichment of *Streptococcus* and *Megasphaera*, which are also enriched in paired airway microbiota (highlighted in green text), and depletion of microbial genera typically associated with health. Mean differences in reads/sample are plotted for the top differentiating taxa (genus-level identity provided). Significance was set at a false discovery rate *q* < 0.05, read difference ≥ 100 reads, and presence in ≥ 50% of the enriched group. OTU, operational taxonomic unit

Since CD4 status was related to gut microbiota composition, and CD4 depletion is associated with more severe disease and epithelial barrier dysfunction, we postulated that patients with lower CD4 counts would exhibit a higher degree of compositional similarity between their airway and gut microbiota, indicative of microbial translocation between these two body habitats. To examine this, patients were stratified based on CD4 cell count (high versus low) and the proportion of those with low or high airway–gut microbiota P-D was compared. As hypothesized, patients with low CD4 counts exhibited significantly smaller gut–airway paired distances indicating a greater degree of microbiota compositional similarity between these two sites (low P-D; Fisher’s exact test, *p* < 0.001; Fig. 4a). To identify taxa that discriminated CD4-low and CD4-high patient stool microbiota, distribution modeling was performed and validated using random forest analysis (Additional file 1: Figure S8). The stool of HIV-infected patients with high CD4 counts were enriched for traditional gastrointestinal genera such as *Prevotella*, *Faecalibacterium*, *Bacteroides*, and *Ruminococcus* (Fig. 4b), none of which were detected in any paired BAL sample of these patients. In contrast, those patients with low CD4 counts exhibited enrichment of specific *Veillonella*, *Lactobacillales*, *Streptococcus*, and *Megasphaera* taxa, the latter two of which were also detected in paired BAL of these patients, implicating translocation and systemic infection by *Streptococcus* and *Megasphaera* in the most severely ill HIV-infected patients (Fig. 4b).

Using stool samples from an independent cohort of Ugandan, HIV-infected and uninfected patients without pneumonia, previously examined by Monaco et al [14], we validated these taxonomic enrichments. Consistent with our observations, HIV-infected patients with the highest CD4 counts within the Monaco study were enriched for members of *Prevotella*, *Ruminococcaceae*, and *Bacteroides*, whereas those with the lowest CD4 counts were enriched for members of *Streptococcus*, *Lactobacillus*, and *Veillonella* (Additional file 1: Table S4). When only untreated HIV-infected patients were compared to HIV-uninfected patients, *Veillonella* was again enriched in the HIV-infected population (Additional file 1: Table S5). Thus, specific gut microbiota taxonomic perturbations associated with CD4 status are consistent irrespective of pneumonia or antiretroviral treatment status in HIV-infected patient populations.

### Gut microbial products differentially modulate macrophage phenotype based on patient CD4 count

**Fig. 5:**
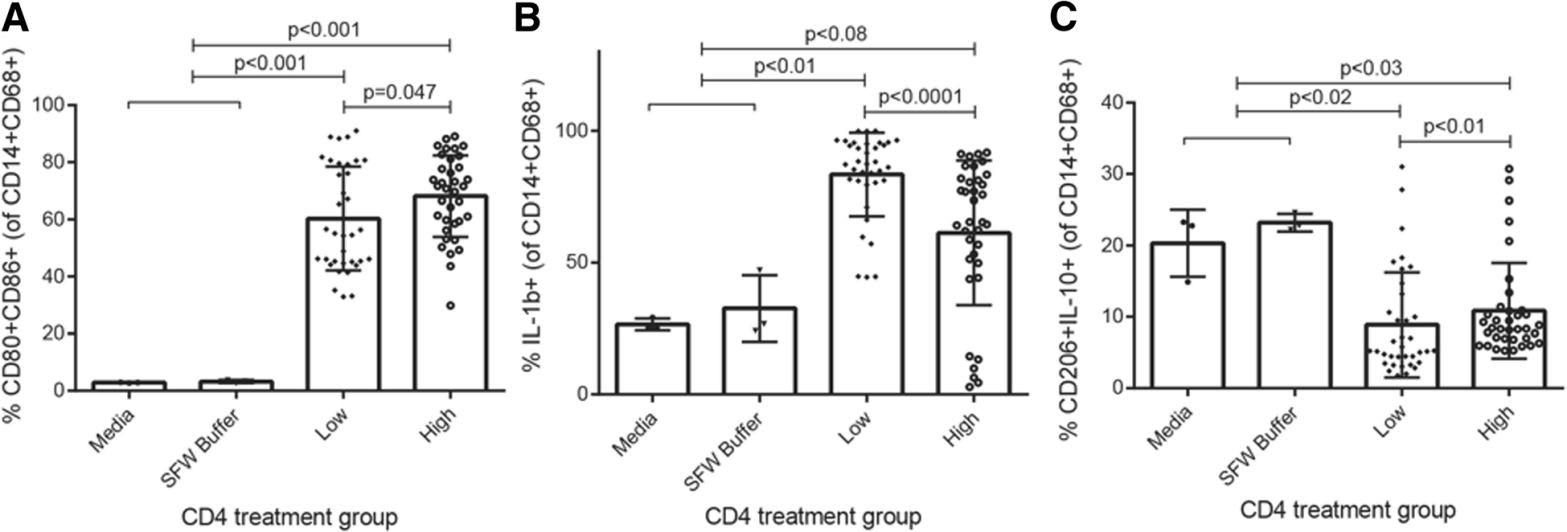
Sterile fecal water from patients with low CD4 counts reduces macrophage activation, increases IL-1β expression, and decreases the frequency of tissue repair macrophages. **a** Compared with CD4-high patients, sterile fecal water from CD4-low patients induces fewer activated CD80+ CD86+ THP-1-derived macrophages (KW, *p* < 0.001; all treatments versus media or buffer *p* < 0.001). **b** Increased pro-inflammatory IL-1β+ THP-1-derived macrophages are induced by CD4-low compared to CD4-high patient SFW (KW, *p* < 0.0001; low or intermediate versus media or buffer *p* < 0.01, high versus media or buffer *p* < 0.08). **c** CD4-low patient sterile fecal water decreased tissue repair associated CD206+ IL-10+ THP-1-derived macrophages (compared with CD4-high patients; KW, *p* < 0.001, low versus media or buffer *p* < 0.05, intermediate or high versus media or buffer *p* < 0.06). Low and high subgroups are CD4 count quartiles 1 and 4, respectively. Data representative of two independent experiments; 24 biological replicates with three technical replicates each are plotted. Plotted *p* values from Mann–Whitney test. KW, Kruskal–Wallis; SFW, sterile fecal water

Peripheral monocyte-derived macrophages are necessary for control of lower airway microbial infection and for resolution of inflammation in pneumonia patients. However, macrophage dysfunction is a hallmark of advanced HIV infection and commonly characterized by decreased circulating monocytes and increased pro-inflammatory macrophages within the gut [15]. In HIV-infected patients, pneumonia is not only more common, it is also frequently fatal due to rampant, systemic inflammation and failure to control microbial infection. More recently, it has been demonstrated that the gut microbiome modulates airway immunity and response to respiratory infection [2–4, 16]. We therefore hypothesized that within the most severely ill HIV-infected patients, gut microbiota-associated products promote a pro-inflammatory macrophage effector phenotype with reduced capacity for tissue repair, contributing to innate immune dysfunction and failure to control infection. To investigate this, the THP-1 macrophage cell line was differentiated for 2 days with phorbol 12-myristate 13-acetate (PMA) prior to 24-h treatment with sterile fecal water (SFW, see the “Materials and methods” section) from patients with either the highest or lowest CD4 counts to assess the effect of products of the gut microbiome on macrophage effector function. Following SFW treatment, THP-1 cells were assessed via flow cytometry for differentiation (CD14+CD68+), activation (CD80+CD86+), pro-inflammatory (IL-1β+), and tissue repair (CD206+IL-10+) markers. While treatment with SFW derived from CD4 low subjects resulted in significantly fewer activated macrophages compared with CD4 high subjects (Mann–Whitney; CD4 *p* = 0.047, Fig. 5a), a significantly larger proportion of THP-1 cells were pro-inflammatory IL-1β+ macrophages (*p* < 0.0001, Fig. 5b), and fewer exhibited capacity for tissue repair (*p* < 0.01, Fig. 5c; see Additional file 1: Figure S9 for gating strategy). These observations also held true when samples were stratified by P-D, which we consider a surrogate for the degree of microbial translocation in these patients (Additional file 1: Figure S10A-C). Hence, the associated products of HIV-infected pneumonia patient stool, the majority of which (70–80% by stool volume) are microbial-derived, differentially modulate macrophage activation and differentiation based on patient disease severity and degree of microbial translocation. These data indicate that changes in the gut microbiota associated with more severe immune suppression in HIV-infected pneumonia patients may have meaningful consequences for macrophage dysfunction and respiratory infection outcomes.

## Discussion

The biological factors that determine disease severity and outcome in HIV-infected patients with pneumonia are incompletely understood, and prior studies have intuitively focused on the role of the lower airway microbiota [1, 17, 18]. Previous investigations of the HIV–pneumonia lower airway microbiome have described distinct microbiota composition states dominated by *Prevotellaceae*, *Streptococcaceae*, or *Pseudomonadaceae* (a member of the *Gammaproteobacteria*) which differentially relate to airway immune response profile in these patients, but not to peripheral immune activation status [1]. This lack of relationship between airway microbiota and systemic immunity has also been described previously in cohorts of healthy subjects [1, 19]. Our study confirms earlier observations noting the existence of three compositionally distinct lower airway microbiota in HIV-infected pneumonia patients, each dominated by a member of the *Prevotellaceae*, *Streptococcaceae*, or *Gammaproteobacteria*. Also consistent with previous findings was the observation that *Prevotellaceae* and *Streptococcaceae* dominated communities possessed similar co-associated microbial taxa that differ primarily in their relative distribution. Both of these microbiota states were also significantly more diverse and evidenced a higher degree of fungal presence compared with the *Gammaproteobacteria*-dominated communities. However, as we have previously found, the airway microbiota is not related to the degree of disease severity (as defined by CD4 cell counts), leading us to consider a role for the gut microbiome in modulating peripheral immune function and response to bacterial pneumonia in these patients.

A growing body of evidence implicates the gut microbiome in modulating local and remote mucosal immunity, as well as systemic markers of inflammation in response to microbial infection [3, 6, 16, 20]. Here, we provide evidence that while both airway and gut microbiota are perturbed in parallel in HIV-infected pneumonia patients, it is not the airway, but rather the gut microbiota that relates to peripheral CD4 cell counts. Microbial translocation is an established phenomenon within severely ill HIV-infected individuals who exhibit CD4+ T cell depletion and mucosal barrier breakdown. We show that the degree of immune suppression in our large cohort correlates with increased gut–airway microbiota similarity—a surrogate marker of systemic bacterial infection. HIV-infected pneumonia patients with lower CD4 counts exhibit a greater degree of shared bacterial taxonomy in their gut and airway microbiota, and specific gut-enriched taxa belonging to *Streptococcus* and *Veillonella* are also detected in the airways of these more severely ill patients, indicating that systemic infection may be perpetrated by a relatively small number of specific genera. These findings are pertinent in light of our re-analysis of the Monaco et al. cohort which indicates that gut microbiome enrichment of *Streptococcus* and *Veillonella* in HIV-infected patients with low CD4+ cell counts is more generalizable and occurs irrespective of pneumonia or HIV treatment, and may be indicative of traditional airway-colonizing microbes establishing a niche within the gut or *vice versa*.

Monocyte-derived macrophages are critical to resolution of lower airway infection and inflammation [21, 22], particularly in HIV-infected patients with reduced capacity to mount an adaptive immune response. Progressive HIV infection is known to result in depletion of circulating monocytes and accumulation of poorly activated, pro-inflammatory macrophages within the gut [15, 23]. Independently, it has been established that the gut microbiome modulates local and systemic immunity via microbial-derived products [3, 4, 16, 24] and that gut microbiome dysbiosis is a hallmark of HIV infection [6, 9, 14]. We therefore investigated whether sterile, fecal microbial products from HIV-infected pneumonia patients differentially modulate macrophage function in a manner consistent with the immunosuppressive status of patients in our study. Human stool is primarily composed of microbes (70–80%); thus, sterile fecal water (SFW) is a reliable source of gut microbial products and is primarily derived of microbial-derived ligands and metabolites, with the remaining material made up of host- or food-derived products. Using the THP-1 cell line, we demonstrate that SFW from patients with low CD4 counts is less capable of promoting activated (CD80+CD86+) or tissue repair (CD206+IL-10+) macrophages compared to CD4-high patient SFW and instead induces pro-inflammatory (IL-1β+) macrophages. These results indicate that at least in vitro, the products of the gut microbiome from HIV-infected pneumonia patients modulate macrophage activation and differentiation in a disease severity-dependent manner. Although our study was unable to identify a role for the airway microbiota in this response, it is still unknown whether gut microbial products work in concert with airway microbial products to induce immune responses and whether increased sharing of microbes between the lungs and gut is due to translocation from the gut to the airways, *visa-versa*, or an independent source. Nonetheless, it is plausible that the products of the gut microbiome of CD4-low patients program pro-inflammatory macrophage effector phenotypes in vivo, contributing to the failure to control microbial infection. However, follow-up studies are necessary to prove whether manipulation of the gut microbiota independent of the airways can improve pneumonia outcomes.

Limitations of this study include its cross-sectional nature and lack of pre-pneumonia paired airway and stool samples, the latter of which is logistically difficult to obtain in this patient population. It is also important to note that limited antibiotic and medical history is available for this patient population. Similar to standard practice in the USA, HIV-infected individuals hospitalized with suspected pneumonia in Uganda are invariably treated with empiric antibiotics while the diagnostic evaluation is in progress and a recommended first-line antibiotic for hospitalized, non-ICU patients is ceftriaxone. The vast majority (> 93%) of patients in this study received ceftriaxone during this study; however, we were unable to obtain accurate information on prior antibiotic use due to lack of medical records in this population. Despite these limitations, this study is the first to reveal a systemic microbiota perturbation in HIV-infected pneumonia patients and a relationship between gut microbiota composition and CD4 status. Moreover, it provides the first evidence that the products of the gut microbiome of HIV-infected patients with low CD4 counts alter monocyte effector phenotypes, reducing their capacity for repair and promoting a program of pro-inflammatory activity. These investigations add to the growing body of work implicating the gut microbiome in airway disease and infection outcomes and suggest that novel therapeutic strategies that focus on both body habitats may lead to improved outcomes in HIV patients with acute respiratory infection.

## Materials and methods

### Study design

We enrolled subjects infected with HIV admitted to Mulago Hospital in Kampala, Uganda, for acute pneumonia from June 2012 to November 2015 as part of the Lung MicroCHIP (Lung Microbiome in Cohorts of HIV-Infected Persons) study. Patients underwent two sputum acid-fast bacilli (AFB) smear examinations to diagnose pulmonary TB. Patients who were sputum AFB smear negative underwent bronchoscopy with bronchoalveolar lavage (BAL) for clinical diagnosis, as previously described [1], with 10 ml set aside for microbiome analysis. BAL was performed in the lung segment that was most involved on chest radiograph. Bronchoscopy was performed a median of 1 day after hospital admission (interquartile range, 1–3 days) with concurrent collection of stool. Clinical data were collected and diagnoses were assigned as previously described [18]. Study endpoints were hospital discharge and 2-month vital status.

### Ethics statement

The Makerere University School of Medicine Research Ethics Committee, the Mulago Hospital Research and Ethics Committee, the Uganda National Council for Science and Technology, and the University of California San Francisco Committee on Human Research approved the protocol. Subjects provided written informed consent.

### Bacterial and fungal community profiling

#### DNA extraction

Individual BAL and stool samples were placed into lysing matrix E (INTEGRA Biosciences) tubes pre-aliquoted with 500 μl of hexadecyltrimethylammonium bromide (CTAB; Sigma Aldrich) DNA extraction buffer and incubated at 65 °C for 15 min. An equal volume of phenol:chloroform:isoamyl alcohol (25:24:1; Sigma Aldrich) was added to each tube, and samples were homogenized in a Fast Prep-24 homogenizer at 5.5 m/s for 30 s. Tubes were centrifuged for 5 min at 16,000×*g*, and the aqueous phase was transferred to individual wells of a deep-well 96-well plate. An additional 500 μl of CTAB buffer was added to the LME tubes, the previous steps were repeated, and the aqueous phases were combined. An equal volume of chloroform was added to each sample and mixed followed by centrifugation at 3000×*g* for 10 min to remove excess phenol. The aqueous phase (600 μl) was transferred to a deep-well 96-well plate, combined with two volume equivalents of polyethylene glycol (PEG) and stored overnight at 4 °C to precipitate DNA. Plates were centrifuged for 60 min at 3000×*g* to pellet DNA, and the PEG solution was removed. DNA pellets were washed twice with 300 μl of 70% ethanol, air-dried for 30 min, and suspended in 100 μl of sterile water. DNA samples were quantitated using the Qubit dsDNA HS Assay Kit and diluted to 10 or 2 ng/μl for stool or BAL, respectively. No template controls (NTCs, one per extraction plate) were processed similar to samples.

#### 16S DNA amplification and sequencing

The V4 region of the 16S rRNA gene was amplified in triplicate, as previously described [4]. Triplicate reactions each possessed one no-template control to assess background contamination. Triplicate reactions were combined and purified using the SequalPrep Normalization Plate Kit (Invitrogen) according to the manufacturer’s specifications. Purified amplicons were quantitated using the Qubit dsDNA HS Assay Kit and pooled at equimolar concentrations. For NTCs, total volume was pooled because burden was too low for equimolar concentrations. The amplicon library was concentrated using the Agencourt AMPure XP system (Beckman–Coulter) quantitated using the KAPA Library Quantification Kit (KAPA Biosystems) and diluted to 2 nM. Equimolar PhiX was added at 40% final volume to the amplicon library and sequenced on the Illumina NextSeq 500 Platform on a 153 bp × 153 bp sequencing run.

#### ITS2 DNA amplification and sequencing

The internal transcribed spacer region 2 (ITS2) of the rRNA gene was amplified in triplicate, as previously described [4]. Amplification and sequencing were performed with the same protocols as above with the following modifications: PhiX (5 pM final) to the amplicon library and sequenced on the Illumina MiSeq Platform on a 290 bp × 290 bp sequencing run.

#### 16S OTU table generation

Raw sequence data was converted from bcl to fastq format using bcl2fastq v2.16.0.10. Paired sequencing reads with a minimum overlap of 25 bp were merged using FLASH v1.2.11. Successfully merged reads were identified, had index sequences extracted, and were demultiplexed in the absence of quality filtering using QIIME (Quantitative Insights Into Microbial Ecology, v1.9.1). Reads were then quality filtered using USEARCH’s fastq filter (v7.0.1001) to remove reads having > 2 expected errors. Following this step, BAL and stool samples were both processed individually, as well as part of a combined dataset. Quality-filtered reads were dereplicated at 100% identity, clustered at 97% sequence identity into operational taxonomic units (OTUs) if they had ≥ 2 reads and had chimeras removed, and mapped back to the resulting OTUs using USEARCH v8.0.1623. The Greengenes database (May 2013) was used to assign taxonomy to OTUs, and QIIME was used to make the phylogenetic tree. OTUs were filtered by (1) removing any known common contaminant OTU present in more than half of the negative extraction controls for this study and (2) removing any remaining OTU that had a total read count across all samples less than 1/5000th of a percent of the total read counts across all samples for total OTU picking, or removing any OTU with less than 70 reads for BAL samples and less than 250 reads for stool samples. Finally, sequencing reads were representatively normalized by rarefying 100 times and using sample centroids as described previously [4]. Total sample OTU picking was rarefied to 51,997, BAL alone to 67,135, and stool alone to 120,665 reads per sample. Total reads from each NTC were below the level of rarefying, and NTCs for BAL samples possessed a maximum of 2 reads/NTC at dereplication. Raw sequences from 110 Ugandan HIV-infected and uninfected individuals’ stool samples previously examined by Monaco et al. [14] were processed in accordance with this pipeline. OTUs containing less than 1/1000th of a percent of total reads were removed, and remaining reads were rarefied to 83,041 reads/sample, resulting in 99 samples retained.

#### Controlling for low biomass samples

Airway samples (e.g., BAL) are known to possess significantly lower bacterial biomass than stool samples in healthy individuals; to control for the potential of higher extraction, PCR, and sequencing contamination in these and in stool samples, several steps were taken. (1) Every extraction batch had a minimum of one negative control (PBS) per plate, placed in the middle of the plate. These control extractions were taken through the full sequencing pipeline. (2) PCR for each sample was performed in triplicate alongside a negative control (H_2_0). Any PCR with a band in the negative control was rerun. (3) Negative controls were carried forward with maximum volume possible at each step of the sequencing pipeline. All negative controls for BAL samples with the exception of one failed to sequence. The one BAL negative control that sequenced possessed two reads, neither of which made it through quality filtering of the data nor belonged to an OTU present in real samples. (4) As a final stringency, low burden OTUs were removed from all samples (see the “Materials and methods” section) as a denoising step.

#### ITS2 OTU table generation

ITS2 OTU tables were generated using the same protocol as above with the following modifications and notes: (1) Chimeras were removed, and taxonomy was assigned using the UNITE database (January 2016, [25]); (2) no phylogenetic tree was generated; (3) no NTC successfully sequenced; (4) any OTU was removed if it possessed a total read count less than 1/5000th of a percent of the total read counts across all samples for total, BAL, or stool OTU picking; and (5) samples were representatively rarefied to 1000, 1044, or 2565 reads/sample for total, BAL, or stool OTU picking, respectively.

### THP-1 sterile fecal water assay and flow cytometry

Stool samples from 12 CD4-low patients and 12 CD4-high patients were used as biological replicates and also stratified based on Bray–Curtis paired distance into 6 low, 11 intermediate, and 7 high samples. Samples were chosen based on CD4-low or CD4-high status and sufficient material. Stool samples were homogenized 1 g ml^−1^ (*w*:*v*) in pre-warmed phosphate-buffered saline (PBS) containing 20% heat-inactivated fetal bovine serum (FBS). Samples were vigorously vortexed, incubated (37 °C water bath, 10 min), and centrifuged (14,000×*g*, 10 min). Supernatant was removed to a new tube and centrifuged again (16,000×*g*, 1 h). Supernatant was filter-sterilized through a 0.4-μm filter, followed by a 0.2-μm filter, before sterile fecal water (SFW) was used in the THP-1 assay described below. SFW buffer (PBS with 20% FBS) and R10 media [Roswell Park Memorial Institute media 1640 with 10% heat-inactivated FBS (antigen activator) and 2 mM L-glutamine and 100 U ml^−1^ penicillin–streptomycin added; Life Technologies] were used as the negative controls.

The THP-1 monocyte cell line (passages 2–3) was cultured for 48 h with 10 ng/ml Phorbol 12-Myristate 13-Acetate (PMA, Thermo Fisher Scientific) added to R10 media to induce macrophage differentiation. Half of the culture media was changed to new R10 with SFW (final SFW concentration 8%) for 24 h. Controls included R10 and SFW buffer. To assess cytokine production, the cultures were mixed with GolgiPlug (Gplug; BD Biosciences) for 12 h before staining for flow cytometry. Experiment was replicated twice due to fecal material scarcity, with three technical replicates per biological replicate.

For flow cytometry, single-cell suspensions were stained using a panel of antibodies, including anti-CD14 (63D3, 1:100; BV711, BioLegend), anti-CD68 (Y1/82A, 1:40; PE-Cy7, BioLegend), anti-CD80 (2D10, 1:50; FITC, BioLegend), anti-CD86 (IT2.2, 1:50; BV650, BioLegend), anti-CD206 (15-2, 1:50; APC-Cy7, BioLegend), anti-IL-1β (H1b-98, 1:20; Pacific Blue, BioLegend), and anti-IL-10 (JES3-9D7, 1:20; PE, Miltenyi Biotec). Validation for each primary antibody is provided on the manufacturers’ websites. Dead cells were stained positive with LIVE–DEAD Aqua Dead Cell Stain (Life Technologies). Permeabilization buffer (BD Biosciences) was used to permeabilize cells before staining for the intracellular markers CD68, IL-1β, and IL-10. For flow analysis, live THP-1 macrophages were gated as CD14^+^CD68^+^ cells. Among macrophage subpopulations, activated cells were CD80^+^CD86^+^, pro-inflammatory cells were IL-β^+^, and tissue repair cells were both CD206^+^ and IL-10^+^. Stained cells were assayed via a flow cytometer on a BD LSR II (BD Biosciences).

### Statistical analysis

Diversity and richness indices were calculated using QIIME. Distance matrices [weighted UniFrac [26] for BAL comparison with previous lung studies; Bray–Curtis for stool and shared taxa] were calculated in QIIME [27] to assess compositional dissimilarity between samples and visualized using PCoA plots constructed in Emperor [28]. Bray–Curtis paired distance was determined using whole dataset OTU picking (see Additional file 1: Supplemental Methods for code, [29]). Permutational multivariate analysis of variance (PERMANOVA) was performed using *Adonis* in the R environment (*vegan* package) to determine factors that explained variation in microbiota composition (see Additional file 1: Supplemental Methods for code) and was used to confirm that BAL and stool composition were not related to processing factors (*p* ≥ 0.1). Each clinical, microbiological, and demographic factor was initially examined via PERMANOVA in relationship with microbial composition. PERMANOVA examines each variable independently of all other variables examined and does not require additional adjustment against other factors being examined [30]. We then further examined only those factors which were statistically significant within PERMANOVA. Unsupervised hierarchical clustering was performed using the *hclust* function in the R environment (*stats*, *heatmap3* packages) with Bray–Curtis distance (*vegan* package) and Ward 2 cluster method on bacterial families present in ≥ 1 sample at ≥ 3% abundance.

To determine which OTUs differed in relative abundance between patient CD4, paired distance, or infection groups, the distribution of each OTU was modeled using Poisson, negative binomial, or zero-inflated negative binomial distributions and the best fit distribution was assigned based on Akaike information criterion (AIC), as previously described [12]. This modeling approach is appropriate for sequence-count data, and was corrected for multiple testing using false-discovery rate [*q <* 0.05, see Additional file 1: Supplemental Methods for code, [31]]. Results were presented if ≥ 50% of the enriched group possessed the OTU and mean OTU difference between patient groups was ≥100 reads/sample. Random forest classification was performed in the R environment with package *randomForest* using 5000 trees to validate OTU distribution modeling (see Additional file 1: Supplemental Methods for code).

When examining the association between clinical or microbiological factors and patient groups, *p* values were calculated on the basis of covariate distribution by Kruskal–Wallis (numerical, skewed distribution, > 2 groups), Mann–Whitney (numerical, skewed, 2 groups), chi-square (categorical), or Fisher’s exact (sparse categorical) tests. To test for THP-1 differences based on patient group, Kruskal–Wallis was performed in GraphPad Prism 6. Except where indicated, all analyses were conducted in the R statistical programming environment.

## Additional file

Additional file 1: Supplemental Methods: Rscripts. **Figure S1.** DESEq2 normalization confirms body habitat specificity of stool and BAL bacterial communities. **Figure S2.** Lower airway fungal communities are primarily dominated by *Candida*. **Figure S3.** Similar to lower airways, stool of HIV-infected pneumonia patients is consistently dominated by *Candida* and does not show anatomic site distinction from the lower airways. **Figure S4.** The antibiotic ceftriaxone significantly explains a minor proportion of variance in stool bacterial microbiota. **Figure S5.** Stool bacterial microbiota is related to circulating CD4 count. **Figure S6.** Stool bacterial microbiota composition differs based on similarity to paired BAL sample. **Figure S7.** BAL and stool dominant bacterial family do not relate to one another within patients. **Figure S8.** Random Forest confirms CD4 and paired distance associated enrichments. **Figure S9.** THP-1 flow gating strategy. **Figure S10.** Sterile fecal water from patients with low P-D induce increased activated and pro-inflammatory macrophages and decreased tissue repair macrophage differentiation. **Table S1.** Clinical and demographic features of the HIV-infected pneumonia patient cohort. **Table S2.** Clinical features across airway microbiota. **Table S3.** HIV-infected pneumonia patient stool fungal composition is consistently dominated by *Candida* or *Saccharomyces*. **Table S4.** Taxa relatively enriched in the gut microbiota of CD4 low (white) versus CD4 high (gray) HIV-infected patients (based on first and fourth quartile respectively; Monaco Study). **Table S5.** Taxa relatively enriched in the gut microbiota of HIV uninfected (white) versus HIV infected (gray) subjects (Monaco study). (DOCX 6354 kb)
