# Supplementary material for: Gut microbiota in HIV–pneumonia patients is related to peripheral CD4 counts, lung microbiota, and in vitro macrophage dysfunction
Source: Microbiome. 2019 Mar 11;7:37. doi: 10.1186/s40168-019-0651-4 (PMC6413461; doi:10.1186/s40168-019-0651-4)
Supplement: Supplementary file 1 — Supplemental Methods: Rscripts. Figure S1. DESEq2 normalization confirms body habitat specificity of stool and BAL bacterial communities. Figure S2. Lower airway fungal communities are primarily dominated by Candida. Figure S3. Similar to lower airways, stool of HIV-infected pneumonia patients is consistently dominated by Candida and does not show anatomic site distinction from the lower airways. Figure S4. The antibiotic ceftriaxone significantly explains a minor proportion of variance in stool bacterial microbiota. Figure S5. Stool bacterial microbiota is related to circulating CD4 count. Figure S6. Stool bacterial microbiota composition differs based on similarity to paired BAL sample. Figure S7. BAL and stool dominant bacterial family do not relate to one another within patients. Figure S8. Random Forest confirms CD4 and paired distance associated enrichments. Figure S9. THP-1 flow gating strategy. Figure S10. Sterile fecal water from patients with low P-D induce increased activated and pro-inflammatory macrophages and decreased tissue repair macrophage differentiation. Table S1. Clinical and demographic features of the HIV-infected pneumonia patient cohort. Table S2. Clinical features across airway microbiota. Table S3. HIV-infected pneumonia patient stool fungal composition is consistently dominated by Candida or Saccharomyces. Table S4. Taxa relatively enriched in the gut microbiota of CD4 low (white) versus CD4 high (gray) HIV-infected patients (based on first and fourth quartile respectively; Monaco Study). Table S5. Taxa relatively enriched in the gut microbiota of HIV uninfected (white) versus HIV infected (gray) subjects (Monaco study). (DOCX 6354 kb) [file 40168_2019_651_MOESM1_ESM.docx]

**ONLINE DATA SUPPLEMENTAL METHODS AND FIGURES**

**Gut microbiota in HIV-pneumonia patients is related to peripheral CD4 counts, lung microbiota and *in vitro* macrophage dysfunction**

Meera K. Shenoy^1, 2*^, Douglas W. Fadrosh^1^, Din L. Lin^1^, William Worodria^3^, Patrick Byanyima^3^, Emmanuel Musisi^3^, Sylvia Kaswabuli^3^, Josephine Zawedde^3^, Ingvar Sanyu^3^, Emily Chang^4, 5^, Serena Fong^4^, Kathryn McCauley^1^, J. Lucian Davis^6^, Laurence Huang^4, 5+^, and Susan V. Lynch^1+^

**Supplemental Methods: Rscripts**

*PERMANOVA performed on distance matrix to determine whether variation in specific variables of interest can be explained by the microbiota, and the percentage of this variation that is explained.*

#Code to Run Adonis from Distance Matrix or OTU table

#Code CANNOT compute PERMANOVAs from OTU tables for Weighted or Unweighted UniFrac.

#K. McCauley, Lynch lab.

rm(list=ls()) #Clean the working environment

library(vegan) #initialize the 'vegan' package (you may need to install it first: it won't initialize if it's not downloaded)

library(parallel) # allows parallelization

loopAdonis <- function(skipcol,datafile,metadata,workdir,use_OTUtable=FALSE, method="bray", perms=1000,sampvar="#SampleID", save=FALSE, outputfile,cores=1,skip_first_line=TRUE,verbose=TRUE) {

#First we set our working directory

setwd(workdir)

#Bring in the distance matrix/OTU table

if(skip_first_line == TRUE) {

dfile <- read.table(datafile,header = T, sep = "\t", check.names = F, comment.char="",row.names=1, skip=1)

} else {

dfile <- read.table(datafile, header=T, sep="\t", check.names=F, comment.char="", row.names=1)

}

#Bring in the metadata

MYmetaEF = read.table(metadata, header = T, sep = "\t", check.names = F,comment.char="") #Changed to check.names=F because I want to keep "#SampleID"

#Create the names of the columns for the output that we will generate

col_names = list("parameter", "R2", "pvalue")

#Create an empty matrix

p = matrix(data=NA, nrow=length(colnames(MYmetaEF))-skipcol, ncol=3, dimnames=list(1:(length(colnames(MYmetaEF))-skipcol), col_names))

#Start the for-loop (going from the first column you are interested in to the last column in the metadata file)

for (i in (skipcol+1):length(colnames(MYmetaEF))){

#Earlier, I tested this script on my PROSE dataset, and the script was taking so long that I wasn't sure it was running

#print("test1")

#Get a list of the ids where the metadata variable isn't missing

ids <- as.character(MYmetaEF[!is.na(MYmetaEF[[i]]),1])

if(use_OTUtable == FALSE) {

sub_MYdat <- subset(dfile, select=ids) # Subset the original untransformed data by selecting samples with no NAs

MYarray2 <- t(sub_MYdat) # Transpose the subset data-matrix

sub_MYdat2 <- subset(MYarray2, select=ids) # Again, select samples with no NAs from the other side of the distance matrix

} else {

sub_MYdat2 <- subset(dfile, select=ids)

}

#print("test2")

set.seed(123) #Make sure your results are reproducible

#Only do this for variables with more than one level

MetaEFSubset <- MYmetaEF[as.character(MYmetaEF[,sampvar]) %in% as.character(ids),] #Create your subset dataset

if(length(table(MetaEFSubset[[i]]))>1) {

if(use_OTUtable == FALSE) {

sub_MYdat2 <- as.dist(sub_MYdat2)

l <- adonis(sub_MYdat2 ~ MetaEFSubset[[i]], data=MetaEFSubset, permutations = perms,parallel=cores) #Adonis from Distance Matrix

} else {

t.MYdat2 <- t(sub_MYdat2)

l <- adonis(t.MYdat2 ~ MetaEFSubset[[i]], data=MetaEFSubset, method=method, permutations=perms, parallel=cores) #If using an OTU table

}

if(verbose==TRUE) {print (l)} #Print out the ADONIS output for i'th variable

m = l$aov.tab #Subset out the section with results that we're interested in

#print("test3")

p[i-skipcol,1] = colnames(MYmetaEF)[i] #print the name of the variable in the first column

p[i-skipcol,2] = m[1,5] #print the R^2 value in the second column

p[i-skipcol,3] = m[1,6] #print the p-value in the third column

if(verbose==TRUE) {print(p)} #print the p data-frame to be kept apprised of progress.

} else {

p[i-skipcol,1] = colnames(MYmetaEF)[i]

}

}

if (save==TRUE) { #If you would like to save your work, you can choose to do so from your function

write.csv(p,file=outputfile) #this writes a csv, and will call it whatever you choose in your function (assuming you use save = TRUE)

}

}

#A simple run of the code

loopAdonis(skipcol=1,datafile="canberra_dm.txt",metadata="mapping.txt",workdir="/data/ ", sampvar="#SampleID", perms=999, save=TRUE, outputfile=" adonis_results.csv", cores=15, use_OTUtable=FALSE, method="bray", skip_first_line=FALSE, verbose=FALSE)

*Representatively rarefy a OTU table so all samples have a single, consensus number of reads per sample to eliminate bias introduced by different numbers of sequences per patient. Samples that do not meet the rarefying threshold are dropped from the dataset.*

####Briefly, many single-rarefied OTU tables are calculated, and the distance between the subject-specific rarefied vectors is calculated. The rarefied vector that is the minimum average (or median) distance from itself to all other rarefied vectors is considered the most representative for that subject and built into the new rarefied table. ####

# Lynch lab.

# library(GUniFrac) # (don't need the package if you call Rarefy below)

library(vegan)

# (Rarefy function from GUniFrac package)

Rarefy <- function (otu.tab, depth = min(rowSums(otu.tab)))

{

otu.tab <- as.matrix(otu.tab)

ind <- (rowSums(otu.tab) < depth)

sam.discard <- rownames(otu.tab)[ind]

otu.tab <- otu.tab[!ind, ]

rarefy <- function(x, depth) {

y <- sample(rep(1:length(x), x), depth)

y.tab <- table(y)

z <- numeric(length(x))

z[as.numeric(names(y.tab))] <- y.tab

z

}

otu.tab.rff <- t(apply(otu.tab, 1, rarefy, depth))

rownames(otu.tab.rff) <- rownames(otu.tab)

colnames(otu.tab.rff) <- colnames(otu.tab)

return(list(otu.tab.rff = otu.tab.rff, discard = sam.discard))

}

#### Parameters ####

# specify the raw OTU count table, with samples = rows, taxa = columns

# rawtab = otu_tab_t

# specify the depth you would like to rarefy your tables to

# the default is to just use the minimum sequencing depth

# raredepth = min(rowSums(rawtab))

# specify the number of rarefied tables you would like to generate

# to calculate your representatiave rarefied table from

# ntables = 100

# specify the distance measure to use to calculate distance between rarefied data sets, for each subject

# can be any of the methods available in the vegdist function of vegan

# distmethod = "euclidean"

# specify the method to summarize across distances

# if mean distance, then summarymeasure = mean

# if median distance, then summarymeasure = median

# summarymeasure = mean

# specify the seed start for the rarefied tables

# for each subsequent table, 1 will be added that the previous seed

# for reproducibility, always save your seedstart value (or just use the default for simplicity).

# seedstart = 500

# specify if you want progress updates to be printed

# verbose = TRUE

### returns a representative rarefied OTU table of class matrix.

reprare <- function(rawtab=otu_tab_t, raredepth = min(rowSums(otu_tab_t)), ntables=100, distmethod="euclidean",

summarymeasure=mean, seedstart=500, verbose=TRUE) {

raretabs = list()

for (z in 1:ntables) {

if (verbose==TRUE) {

print(paste("calculating rarefied table number", z, sep=" "))

}

set.seed(seedstart + z)

raretabs[[z]] = Rarefy(rawtab, depth = raredepth)[[1]]

}

raretabsa = array(unlist(raretabs), dim = c(nrow(raretabs[[z]]), ncol(rawtab), ntables))

final_tab = c()

for (y in 1:nrow(raretabs[[z]])) {

if (verbose==TRUE) {

print(paste("determining rep rarefied vector for subject number", y, sep=" "))

}

distmat = as.matrix(vegdist(t(raretabsa[y,,]), method=distmethod)) # distance across reps for subject y

distsummary = apply(distmat, 2, summarymeasure)

whichbestrep = which(distsummary == min(distsummary))[1] # the best rep is the one with the minimum average/median distance to all other reps. (in case of ties, just select the first)

bestrep = raretabsa[y,,whichbestrep] # select that rep only for subject y

final_tab = rbind(final_tab, bestrep) # build that rep for subject y into final table

}

rownames(final_tab) = rownames(raretabs[[z]])

colnames(final_tab) = colnames(rawtab)

return(final_tab)

}

###### example runs of the function: ######

runexample=FALSE

if (runexample==TRUE) {

### dummy data set for example ###

ntaxa = 200

nsubj = 50

set.seed(444)

dummyOTU <- matrix(sample(0:500, ntaxa*nsubj, prob=c(0.7,0.1,0.1,rep(0.1/498, 498)), replace=TRUE), ncol=ntaxa)

colnames(dummyOTU) = paste("OTU", 1:ntaxa, sep="")

rownames(dummyOTU) = paste("subj", 1:nsubj, sep="")

sort(rowSums(dummyOTU)) # sequencing depth is uneven

# specify the minimum depth

repraretable = reprare(rawtab=dummyOTU, raredepth=min(rowSums(dummyOTU)), ntables=100, distmethod="euclidean",

summarymeasure=mean, seedstart=500, verbose=TRUE)

dim(repraretable)

sort(rowSums(repraretable)) # sequencing depth is now even

# specify a depth other than the minimum

repraretable = reprare(rawtab=dummyOTU, raredepth=3380, ntables=100, distmethod="euclidean",

summarymeasure=mean, seedstart=500, verbose=TRUE)

dim(repraretable) # subjects with less than the minimum are no longer in the table

sort(rowSums(repraretable)) # sequencing depth is now even

}

#repraretable = reprare(rawtab=closed.array, raredepth=min(rowSums(closed.array)), ntables=100, distmethod="euclidean",

#summarymeasure=mean, seedstart=500, verbose=TRUE)

#rep.array<-as.data.frame(t(repraretable))

setwd("/data/")

MYdata=read.table("filtered_otu_table.txt",sep="\t",header=T,comment="",check.names=F)

taxonomy <- MYdata[,c("#OTU ID","taxonomy")]

rownames(MYdata) <- MYdata[,"#OTU ID"]

MYdata[,c("#OTU ID")] <- NULL

MYdata$taxonomy <- NULL

MYarray=as.data.frame(t(MYdata))

repraretable <- reprare(rawtab=MYarray, raredepth=83041, ntables=100, distmethod="euclidean",

summarymeasure=mean, seedstart=123, verbose=TRUE)

rep.array<-as.data.frame(t(repraretable))

#colnames(rep.array)

#head(rep.array)

#head(closed.ureca)

write.table(rep.array,file="/data/filtered_otu_table_MR_83041_raw.txt",sep="\t",quote=F)

#check it

rep.array <- read.table("/data/filtered_otu_table_MR_83041_raw.txt",sep="\t",header=T)

dim(rep.array)

rep.array[,"#OTU ID"] <- row.names(rep.array)

rarefied2 <- merge(rep.array, taxonomy, by=c("#OTU ID"))

write.table(rarefied2,file="/data/filtered_otu_table_MR_83041.txt",sep="\t",quote=F,row.names=F)

*Paired distance: Calculating the distance between two samples collected from distinct anatomic sites from a single patient.*

#### Distance to paired sample ####

#Lynch Lab

rm(list=ls())

setwd("~/data/")

wuf <- "weighted_unifrac_dm.txt"

open.map <- read.table("mapping.txt", header=T, sep="\t",comment="")

dm <- read.table(wuf,header=T,sep="\t",row.names=1)

betadiv.dist <- NULL

obs.count <- table(open.map$SubjectID)

tt <- nrow(obs.count)

for(i in 1:tt) {

id.nm <- names(obs.count[i])

samp.names <- as.character(open.map$X.SampleID[open.map$SubjectID == id.nm])

first.samp <- as.character(samp.names[1])

#If somebody has MORE than one sample (which is expected), do the following

if(sum(samp.names != first.samp)==1) {

newdm <- dm[c(samp.names[1]),c(samp.names[2])]

if(!is.null(newdm)) {

sampid <- names(newdm)

beta.dist.1 <- as.numeric(newdm)

newdm <- cbind(id.nm,beta.dist.1)

#For when there is only one sample

} else {

sampid <- samp.names

newdm <- cbind(id.nm,0)

}

betadiv.dist <- rbind(newdm,betadiv.dist)

}

merged <- merge(open.map,betadiv.dist,by.x="SubjectID",by.y="id.nm")

#adds paired distance to mapping file

write.table(merged, "mapping.txt", sep="\t")

####have to edit table: shift first row over 1, delete first column,

####edit X.SampleID to #SampleID, reorder columns

*Random forest analysis to determine which OTUs predict sample groupings.*

# Script to determine OTUs that predict outcomes

## K. McCauley. Lynch Lab.

workdir <- "/data/ " #Working directory

otutable <- "otutable_16S_rar51997.txt" #OTU table as a text file

mapping_file <- "mapping.txt" #Mapping file

response_var <- "sampleType" #Variable you want to predict

classification <- TRUE # Do you want to use classification or regression

setwd(workdir)

if(readLines(otutable,n=1)=="# Constructed from biom file") {

otu <- read.table(otutable, header=TRUE, sep="\t", skip=1, comment="", check.names=FALSE,row.names=1)

} else {

otu <- read.table(otutable, header=TRUE, sep="\t", comment="", check.names=FALSE,row.names=1)

}

map <- read.table(mapping_file, header=TRUE, check.names=F, comment="", sep="\t",row.names=1)

map <- map[!is.na(map[,response_var]),]

otu2 <- otu[,rownames(map)]

#Need to do some background work to clean up the taxonomies so they aren't long

otunames <- data.frame(OTUname=row.names(otu),tax=otu$taxonomy)

taxanames <- strsplit(as.character(otunames$tax),"; ")

cleantax <- lapply(taxanames, function(x) {

x <- x[-7]

x <- x[which(!x %in% c("g__","f__","o__","c__","p__","k__"))]

x[x != "Unassigned"] <- substring(x[x != "Unassigned"], 4)

x[length(x)]

})

genus_name <- sapply(cleantax, paste, collapse=" ")

otunames$highest_tax <- genus_name

otunames$otulabs <- gsub("_","~",otunames$OTUname)

otunames$fig_names <- paste0(otunames$highest_tax, " (", otunames$otulabs,")")

otu$taxonomy <- NULL

predictors <- t(otu2)

#predictors <- predictors[,apply(predictors, 2, function(x) (sum(x==0)/length(x))<0.80)]

dim(predictors)

response <- map[,response_var]

rf.df <- data.frame(response,predictors)

#Confirms categorical variable

if(classification == TRUE) {

rf.df$response <- factor(rf.df$response)

} else {

rf.df$response <- as.numeric(rf.df$response)

}

pacman::p_load(randomForest)

set.seed(123)

predoutcome <- randomForest(response ~ ., data=rf.df, ntree=5000, importance=TRUE)

print(predoutcome)

plot(predoutcome)

#Plotting the varImp data

imp.df <- as.data.frame(importance(predoutcome))

imp.df <- merge(imp.df, otunames, by.x=0, by.y="OTUname")

imp.df <- imp.df[order(imp.df$MeanDecreaseAccuracy),]

first.30 <- imp.df[(nrow(imp.df)-29):nrow(imp.df),]

first.30$fig_names <- factor(first.30$fig_names,levels=first.30$fig_names)

labs1 <- sapply(strsplit(as.character(first.30$fig_names), " "),

function(x) {

parse(text=paste0("italic('", x[1], "')~", x[2]))

})

library(ggplot2)

a <- ggplot(first.30, aes(y=fig_names, x=MeanDecreaseAccuracy)) + theme_light() + geom_point() + ylab(" ") + ggtitle("Top 30 Predictive OTUs") + scale_y_discrete(labels=labs1)

quartz()

a

*Distribution analysis to determine which OTUs significantly differ between two categories or types of samples.*

#Code to run the three-model OTU Differential-Expression Analysis

#K. McCauley. Lynch lab.

#Before an OTU could go through the model-building process, at least a certain proportion of the cells (here, 25%) had to contain non-zero values. We determined this cutoff by plotting the log of the total reads for each OTU against the proportion of samples with zero-counts. The plot should result in something that looks like a half parabola, in which the cutoff should be the point at which the parabola's edge starts exponentially increasing (Code to make this plot is in the code below, but commented out).

#install.packages(c("lme4","doMC","foreach","MASS","pscl"))

#To install "glmmADMB", see: http://glmmadmb.r-forge.r-project.org/

##Type:

#install.packages("R2admb")

#install.packages("glmmADMB",

# repos=c("http://glmmadmb.r-forge.r-project.org/repos",

# getOption("repos")),

# type="source")

#Set up potentially-used libraries:

require(pscl)

require(MASS)

require(foreach)

require(doMC)

require(lme4)

#require(glmmADMB)

##################

rm(list=ls())

#setwd(".") ## *** IS THIS OK? ***

setwd("/data/ ")

workdir <- "/data/diffExp/" #Directory where you want to save data

otutable <- "otu_table_MR_83k.txt" #OTU table in text file from BIOM format with "taxonomy" as a column

mapfile <- "mapping.txt" #Map file, typically in QIIME format, with "#SampleID" as the first variable

treatment <- "HIV_status" #Variable (from map file) of comparison

trt1 <- "negative" #Reference group in this analysis

trt2 <- "positive"

outputname <- "HIV_status_diffexp.csv" #CSV filename to where results will be sent

subset <- FALSE # Do you want to perform subset analysis? NOTE: if there are too few samples in a group, the models may not respond well, so p-vals/q-vals may be meaningless

ss_var <- NULL # Group you will subset by (i.e., only want the results from a subset of individuals)

ss_group <- NULL # The specific group for which you want results

mixed_effects <- FALSE #Do you want to run a mixed-effects (ME) analysis (ie, theoretically-correlated samples)

ind_id <- NULL #Individual ID: for example "studyID"; Set to NULL if not using ME models otherwise "SubjectID"

drop_warnings <- TRUE #If an OTU's model produces a convergence warning/error, don't include the results -- we're making an assumption that if the OTU doesn't converge, then the resulting coefficient and p-value are not biologically relevant

cores <- 15 #Number of server cores to use

cutoff <- 0.25 #Proportion of samples that must have non-zero values for an OTU to be considered in this analysis

diag_plot <- TRUE

####### Additional Commands

choose_by_BIC <- FALSE #choose the best of the three models using the AIC or BIC. If choose_by_BIC == FALSE, then the script will use the AIC to choose the best model.

skip_first <- FALSE #Skip the first line when reading in your OTU table

###################

# A function for weighted means by each individual

wght_mean_byind <- function(num,id_var) {

dat <- data.frame(num,id_var)

ind.wt <- 1/table(id_var)[table(id_var) != 0]

num.ind <- length(ind.wt)

indwt.df <- data.frame(ind.wt)

wt.dat <- merge(dat,indwt.df,by.x="id_var",by.y=0)

wt.mean <- sum(wt.dat$num*wt.dat$ind.wt)/num.ind

wt.mean

}

# A function to print warnings

myTryCatch <- function(expr) {

warn <- err <- NULL

value <- withCallingHandlers(

tryCatch(expr, error=function(e) {

err <<- e

NULL

}), warning=function(w) {

warn <<- w

invokeRestart("muffleWarning")

})

list(value=value, warning=warn, error=err)

}

###################

#Start Code

#setwd(".")

setwd("/data/ ")

if(skip_first == TRUE) {

MYdata <- read.table(otutable,header = T,sep = "\t", check.names = F, comment.char= "", quote="",row.names=1,skip=1)

} else {

MYdata <- read.table(otutable,header = T,sep = "\t", check.names = F, comment.char= "", quote="",row.names=1)

}

#Our data had headers, was tab separated, used a "#" sign in front of "SampleID", used quotes, and the rownames (ie, OTUIDs) were in the first column and we didn't want to consider them in the data frame as a separate column

taxa.name <- data.frame(row.names(MYdata), as.character(MYdata$taxonomy))

#Separate out the taxonomy from the data, but bring the OTUIDS with the taxonomies (to correctly merge later)

#Drop taxonomy from the MYdata dataframe

MYdata$taxonomy <- NULL

#Read in the map file

MYmeta <- read.table(mapfile,header = T, sep = "\t", check.names = F, comment.char= "")

if(subset == TRUE) {

MYmeta <- MYmeta[MYmeta[,ss_var] == ss_group,]

}

allcols <- length(colnames(MYdata)) #Get the number of OTUs in your data

matrix1.subset <- !is.na(MYmeta[,treatment]) & MYmeta[,treatment]==trt1 #TRUE/FALSE statement for non-missing treatment variable, and being in group 1

matrix1 <- MYdata[c(as.character(MYmeta[,"#SampleID"][matrix1.subset]))] # pull out only the SampleIDs that have data on your variable of interest, based on T/F

matrix2.subset <- !is.na(MYmeta[,treatment]) & MYmeta[,treatment]==trt2 #TRUE/FALSE statement for non-missing treatment variable and being in group 2

matrix2 <- MYdata[c(as.character(MYmeta[,"#SampleID"][matrix2.subset]))] # pull out only the SampleIDs that have data on your variable of interest, based on T/F

matrix3 <- cbind(matrix1,matrix2) #bring two matrices together

mat.array <- t(matrix3) #transform so OTUs are columns

if(mixed_effects == TRUE) {

MYmeta2 <- MYmeta[,c("#SampleID",ind_id,treatment)] #The non-OTU metadata needed for the upcoming loop

both <- merge(mat.array,MYmeta2,by.x=0,by.y="#SampleID") #Add to OTU data

both[,ind_id] <- factor(as.character(both[,ind_id])) #LME models need studyid to be a factor

} else {

MYmeta2 <- MYmeta[,c("#SampleID",treatment)] #The non-OTU metadata needed for the upcoming loop

both <- merge(mat.array,MYmeta2,by.x=0,by.y="#SampleID") #Add to OTU data

}

both$Row.names <- NULL #by.x=0 (above) creates a sudo variable

#Diagnostic Plot for determining acceptable number of non-zero samples per OTU

if(diag_plot == TRUE) {

prev0 <- apply(both[1:length(rownames(MYdata))],2,function(x) {sum(x == 0)/length(x)})

tot.abund <- tot.abund <- apply(both[1:length(rownames(MYdata))],2,function(x) {sum(x)})

pdf("OTU_Cutoff_Diagnostic.pdf")

plot(log(tot.abund),prev0,ylab="Proportion of Samples with Zero Counts",xlab="Log of the Total Reads")

dev.off()

}

registerDoMC(cores)

if(drop_warnings == TRUE) {

options(warn=2) #If the model produces a warning (ie, doesn't converge), then it will treat it like an error and not produce results

}

if(mixed_effects == TRUE) {

usePackages <- c('lme4','glmmADMB')

} else {

usePackages <- NULL

}

all_data <- foreach(i=1:(length(rownames(MYdata))),.combine='rbind',.packages=usePackages) %dopar% {

if(sum(both[,i] != 0)/length(both[,i]) > cutoff) {

OTUname <- colnames(both)[i]

if(mixed_effects == TRUE) {

formula1 <- as.formula(paste0("both[,",i,"] ~ ",treatment," + (1|",ind_id,")"))

formula2 <- as.formula(paste0("both[,",i,"] ~ ",treatment))

if(drop_warnings == TRUE) {

result.pois <- tryCatch(glmer(formula1, family=poisson, data = both),error=function(e) NA)

result.nb <- tryCatch(glmer.nb(formula1, data = both),error=function(e) NA)

result.zinb <- tryCatch(glmmadmb(formula2, random=~1|ind_id, data = both,link="log",family="nbinom",zeroInflation=TRUE),error=function(e) NA)

} else {

pois <- myTryCatch(glmer(formula1, family=poisson, data = both))

nb <- myTryCatch(glmer.nb(formula1, data = both))

zinb <- myTryCatch(glmmadmb(formula2, random=~1|ind_id, data = both,link="log",family="nbinom",zeroInflation=TRUE))

result.pois <- pois$value

result.zinb <- zinb$value

result.nb <- nb$value

warn.pois <- ifelse(is.null(pois$warning),0,as.character(pois$warning))

warn.zinb <- ifelse(is.null(zinb$warning),0,as.character(zinb$warning))

warn.nb <- ifelse(is.null(nb$warning),0,as.character(nb$warning))

error.pois <- ifelse(is.null(pois$error),0,as.character(pois$error))

error.zinb <- ifelse(is.null(zinb$error),0,as.character(zinb$error))

error.nb <- ifelse(is.null(nb$error),0,as.character(nb$error))

}

zinb.coeff <- tryCatch(exp(summary(result.zinb)$coefficients[2,1]),error=function(e) NA)

zinb.pval <- tryCatch(summary(result.zinb)$coefficients[2,4],error=function(e) NA)

aic.zinb <- tryCatch(AIC(result.zinb),error=function(e) NA)

bic.zinb <- tryCatch(AIC(result.zinb,k=log(length(both[,i]))),error=function(e) NA)

nb.disp <- tryCatch(getME(result.nb,"glmer.nb.theta"), error=function(e) NA) # Negative-Binomial dispersion parameter (from NB model)

zinb.disp <- tryCatch(summary(result.zinb)$alpha,error=function(e) NA) #Negative-Binomial dispersion parameter (from ZINB model)

wgt_mean_trt1 <- tryCatch(wght_mean_byind(both[both[,treatment] == trt1,i],both[both[,treatment] == trt1,ind_id]),error=function(e) NA)

wgt_mean_trt2 <- tryCatch(wght_mean_byind(both[both[,treatment] == trt2,i],both[both[,treatment] == trt2,ind_id]),error=function(e) NA)

wgt_mean_diff <- tryCatch((wgt_mean_trt1 - wgt_mean_trt2),error=function(e) NA)

} else {

formula1 <- as.formula(paste("both[,i] ~ ",treatment," | 1",sep=""))

formula2 <- as.formula(paste("both[,i] ~ ",treatment,sep=""))

if(drop_warnings == TRUE) {

result.pois <- tryCatch(glm(formula2, family="poisson", data = both),error=function(e) NA)

result.zinb <- tryCatch(zeroinfl(formula1, data = both, dist = "negbin"),error=function(e) NA)

result.nb <- tryCatch(glm.nb(formula2, data = both),error=function(e) NA)

} else {

pois <- myTryCatch(glm(formula2, family="poisson", data = both))

zinb <- myTryCatch(zeroinfl(formula1, data = both, dist = "negbin"))

nb <- myTryCatch(glm.nb(formula2, data = both))

result.pois <- pois$value

result.zinb <- zinb$value

result.nb <- nb$value

warn.pois <- ifelse(is.null(pois$warning),0,as.character(pois$warning))

warn.zinb <- ifelse(is.null(zinb$warning),0,as.character(zinb$warning))

warn.nb <- ifelse(is.null(nb$warning),0,as.character(nb$warning))

error.pois <- ifelse(is.null(pois$error),0,as.character(pois$error))

error.zinb <- ifelse(is.null(zinb$error),0,as.character(zinb$error))

error.nb <- ifelse(is.null(nb$error),0,as.character(nb$error))

}

zinb.coeff <- tryCatch(exp(summary(result.zinb)$coef$count[2,1]),error=function(e) NA)

zinb.pval <- tryCatch(summary(result.zinb)$coef$count[2,4],error=function(e) NA)

aic.zinb <- tryCatch(AIC(result.zinb),error=function(e) NA)

bic.zinb <- tryCatch(AIC(result.zinb,k=log(length(both[,i]))),error=function(e) NA)

}

mean_trt1 <- tryCatch(mean(both[both[,treatment] == trt1,i]),error=function(e) NA)

mean_trt2 <- tryCatch(mean(both[both[,treatment] == trt2,i]),error=function(e) NA)

mean_diff <- tryCatch((mean_trt1 - mean_trt2),error=function(e) NA)

pois.coeff <- tryCatch(exp(summary(result.pois)$coefficients[2,1]),error=function(e) NA)

pois.pval <- tryCatch(summary(result.pois)$coefficients[2,4],error=function(e) NA)

nb.coeff <- tryCatch(exp(summary(result.nb)$coefficients[2,1]),error=function(e) NA)

nb.pval <- tryCatch(summary(result.nb)$coefficients[2,4],error=function(e) NA)

aic.pois <- tryCatch(AIC(result.pois),error=function(e) NA)

aic.nb <- tryCatch(AIC(result.nb),error=function(e) NA)

bic.pois <- tryCatch(AIC(result.pois,k=log(length(both[,i]))),error=function(e) NA)

bic.nb <- tryCatch(AIC(result.nb,k=log(length(both[,i]))),error=function(e) NA)

variance <- var(both[,i])

trt1vals <- tryCatch(as.numeric(both[,i][which(as.vector(both[,treatment])==trt1)]),error=function(e) NA)

trt2vals <- tryCatch(as.numeric(both[,i][which(as.vector(both[,treatment])==trt2)]),error=function(e) NA)

zerotrt1 <- tryCatch(sum(trt1vals == 0),error=function(e) NA)

zerotrt2 <- tryCatch(sum(trt2vals == 0),error=function(e) NA)

nonzerotrt1 <- tryCatch(length(trt1vals) - zerotrt1,error=function(e) NA)

nonzerotrt2 <- tryCatch(length(trt2vals) - zerotrt2,error=function(e) NA)

totaltrt1 <- tryCatch(sum(trt1vals),error=function(e) NA)

totaltrt2 <- tryCatch(sum(trt2vals),error=function(e) NA)

if(mixed_effects == TRUE) {

if(drop_warnings == TRUE) {

finaldata <- c(OTUname, pois.coeff,pois.pval,nb.coeff,nb.pval,zinb.coeff,zinb.pval,mean_trt1,mean_trt2,mean_diff,wgt_mean_trt1,wgt_mean_trt2,wgt_mean_diff,aic.pois,aic.nb,aic.zinb,bic.pois,bic.nb,bic.zinb,nb.disp,zinb.disp,zerotrt1,zerotrt2, nonzerotrt1, nonzerotrt2, totaltrt1, totaltrt2,variance)

} else {

finaldata <- c(OTUname, pois.coeff,pois.pval,nb.coeff,nb.pval,zinb.coeff,zinb.pval,mean_trt1,mean_trt2,mean_diff,wgt_mean_trt1,wgt_mean_trt2,wgt_mean_diff,aic.pois,aic.nb,aic.zinb,bic.pois,bic.nb,bic.zinb,nb.disp,zinb.disp,zerotrt1,zerotrt2, nonzerotrt1, nonzerotrt2, totaltrt1, totaltrt2,variance,error.pois,error.nb,error.zinb,warn.pois,warn.nb,warn.zinb)

}

} else {

if(drop_warnings == TRUE) {

finaldata <- c(OTUname, pois.coeff,pois.pval,nb.coeff,nb.pval,zinb.coeff,zinb.pval,mean_trt1,mean_trt2,mean_diff,aic.pois,aic.nb,aic.zinb,bic.pois,bic.nb,bic.zinb,zerotrt1,zerotrt2, nonzerotrt1, nonzerotrt2, totaltrt1, totaltrt2,variance)

} else {

finaldata <- c(OTUname, pois.coeff,pois.pval,nb.coeff,nb.pval,zinb.coeff,zinb.pval,mean_trt1,mean_trt2,mean_diff,aic.pois,aic.nb,aic.zinb,bic.pois,bic.nb,bic.zinb,zerotrt1,zerotrt2, nonzerotrt1, nonzerotrt2, totaltrt1, totaltrt2,variance, error.pois,error.nb,error.zinb,warn.pois,warn.nb,warn.zinb)

}

}

}

}

all_data <- data.frame(all_data)

if(mixed_effects == TRUE) {

if(drop_warnings == TRUE) {

names(all_data) <- c("OTUname","pois.coeff","pois.pval","nb.coeff","nb.pval","zinb.coeff","zinb.pval","mean_trt1","mean_trt2","mean_diff","wgt_mean_trt1","wgt_mean_trt2","wgt_mean_diff","aic.pois","aic.nb","aic.zinb","bic.pois","bic.nb","bic.zinb","nb.disp","zinb.disp","zerotrt1","zerotrt2","nonzerotrt1","nonzerotrt2","totaltrt1","totaltrt2","variance")

} else {

names(all_data) <- c("OTUname","pois.coeff","pois.pval","nb.coeff","nb.pval","zinb.coeff","zinb.pval","mean_trt1","mean_trt2","mean_diff","wgt_mean_trt1","wgt_mean_trt2","wgt_mean_diff","aic.pois","aic.nb","aic.zinb","bic.pois","bic.nb","bic.zinb","nb.disp","zinb.disp","zerotrt1","zerotrt2","nonzerotrt1","nonzerotrt2","totaltrt1","totaltrt2","variance","error.pois","error.nb","error.zinb","warn.pois","warn.nb","warn.zinb")

}

} else {

if(drop_warnings == TRUE) {

names(all_data) <- c("OTUname","pois.coeff","pois.pval","nb.coeff","nb.pval","zinb.coeff","zinb.pval","mean_trt1","mean_trt2","mean_diff","aic.pois","aic.nb","aic.zinb","bic.pois","bic.nb","bic.zinb","zerotrt1","zerotrt2","nonzerotrt1","nonzerotrt2","totaltrt1","totaltrt2","variance")

} else {

names(all_data) <- c("OTUname","pois.coeff","pois.pval","nb.coeff","nb.pval","zinb.coeff","zinb.pval","mean_trt1","mean_trt2","mean_diff","aic.pois","aic.nb","aic.zinb","bic.pois","bic.nb","bic.zinb","zerotrt1","zerotrt2","nonzerotrt1","nonzerotrt2","totaltrt1","totaltrt2","variance","error.pois","error.nb","error.zinb","warn.pois","warn.nb","warn.zinb")

}

}

save.image(file=paste0(workdir,imagename,".Rdata"))

colnames(taxa.name) <- c("OTUname","taxonomy")

all_data <- merge(all_data, taxa.name, by=c("OTUname"))

attach(all_data)

if(choose_by_BIC == TRUE) {

result <- t(sapply(seq(nrow(all_data)), function(i) {

best.mod <- c(1,2,3)

bic <- c(as.vector(bic.pois[i]),as.vector(bic.nb[i]),as.vector(bic.zinb[i]))

bic.best <- ifelse(sum(is.na(bic))!=3, best.mod[which(bic == min(as.numeric(bic),na.rm=TRUE))],NA)

mod.name <- c("Poisson","NegBin","ZI-NegBin")[bic.best]

coefs <- c(as.vector(pois.coeff[i]),as.vector(nb.coeff[i]),as.vector(zinb.coeff[i]))

best.coef <- tryCatch(coefs[bic.best],error=function(e) NA)

qvals <- c(as.vector(pois.pval[i]),as.vector(nb.pval[i]),as.vector(zinb.pval[i]))

best.pval <- tryCatch(qvals[bic.best],error=function(e) NA)

list(mod.name,as.numeric(as.character(best.coef)),as.numeric(as.character(best.pval)))

}))

} else {

result <- t(sapply(seq(nrow(all_data)), function(i) {

best.mod <- c(1,2,3)

aic <- c(as.vector(aic.pois[i]),as.vector(aic.nb[i]),as.vector(aic.zinb[i]))

aic.best <- ifelse(sum(is.na(aic))!=3, best.mod[which(aic == min(as.numeric(aic),na.rm=TRUE))],NA)

mod.name <- c("Poisson","NegBin","ZI-NegBin")[aic.best]

coefs <- c(as.vector(pois.coeff[i]),as.vector(nb.coeff[i]),as.vector(zinb.coeff[i]))

best.coef <- tryCatch(coefs[aic.best],error=function(e) NA)

qvals <- c(as.vector(pois.pval[i]),as.vector(nb.pval[i]),as.vector(zinb.pval[i]))

best.pval <- tryCatch(qvals[aic.best],error=function(e) NA)

list(mod.name,as.numeric(as.character(best.coef)),as.numeric(as.character(best.pval)))

}))

}

detach(all_data)

result.mat <- matrix(do.call("rbind",result),ncol=3,nrow=dim(all_data)[1])

colnames(result.mat) <- c("best.mod","best.coef","best.pval")

final.OTUDiff <- data.frame(all_data,result.mat)

final.OTUDiff <- final.OTUDiff[!is.na(final.OTUDiff$best.pval),]

final.OTUDiff$qval.best <- p.adjust(as.vector(final.OTUDiff$best.pval), method="fdr")

setwd(workdir)

write.csv(final.OTUDiff,outputname,row.names=FALSE)

**Figure S1.**

**
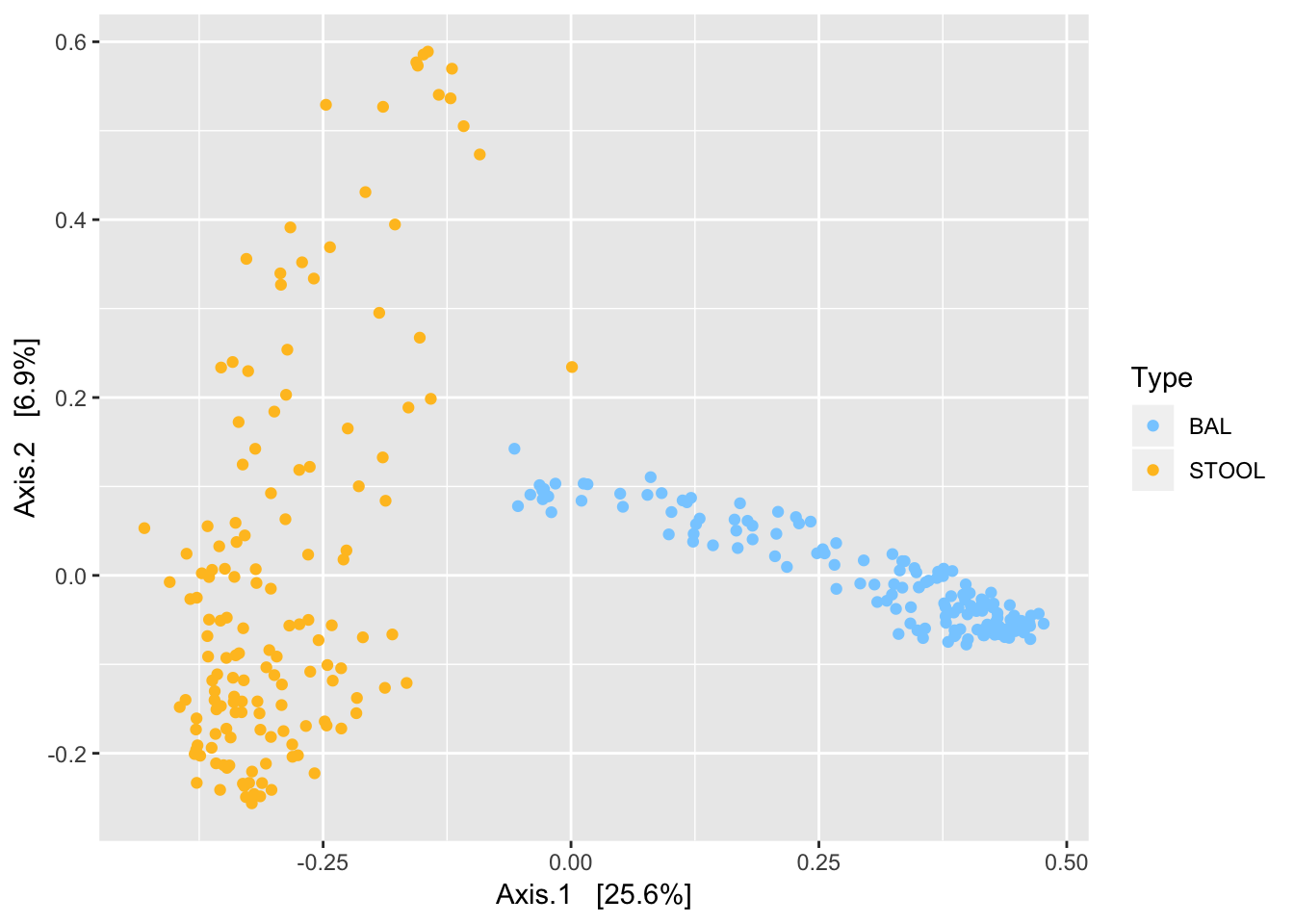
**

**Figure S1. DESEq2 normalization confirms body habitat specificity of stool and BAL bacterial communities.** Principal coordinates analysis (PCoA) using DESEq2 normalization of n = 291 bronchoalveolar lavage (BAL) and stool bacterial community profiles.

**Figure S2.**


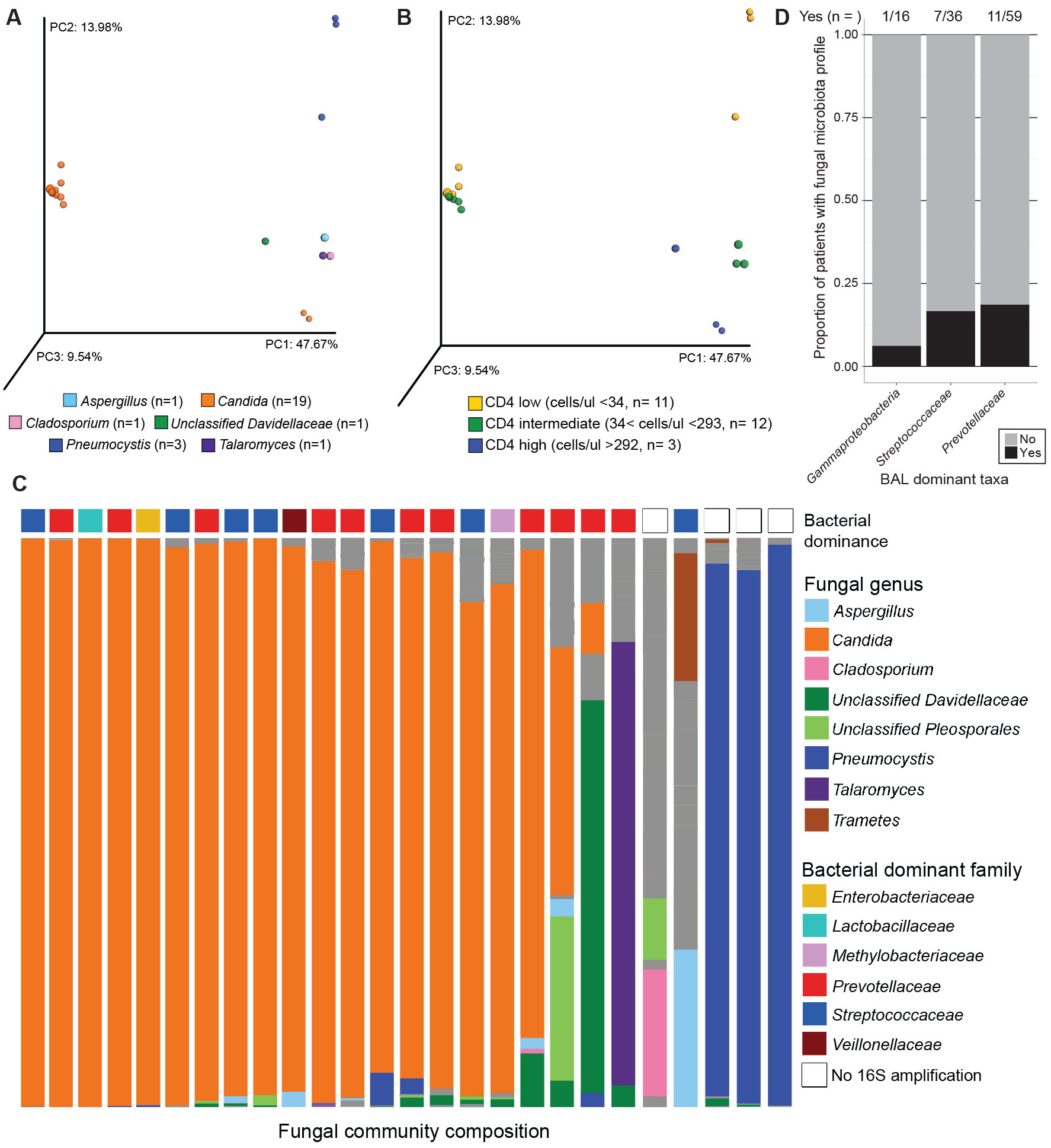

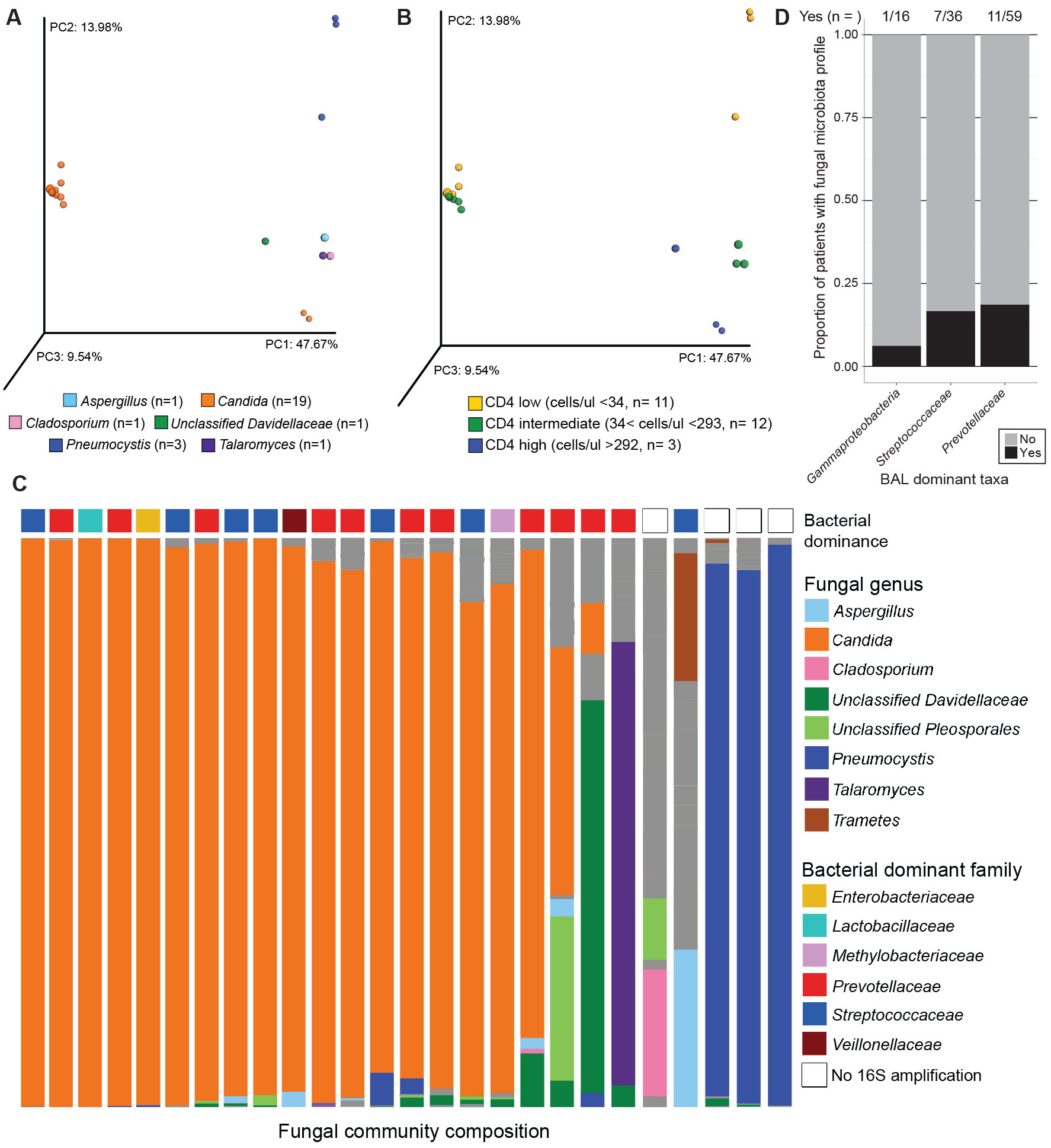

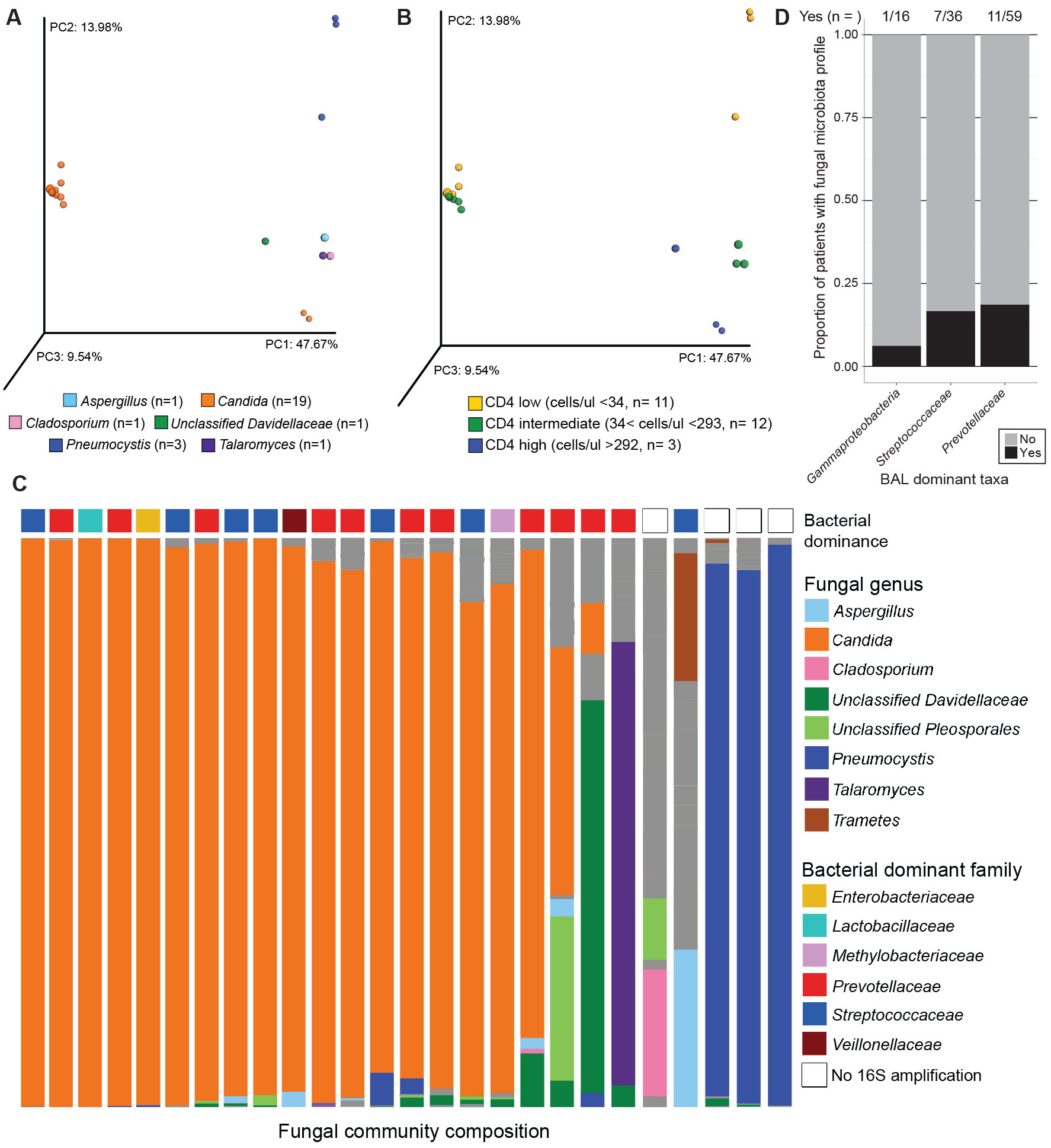


A

B

C

D

**Figure S2. Lower airway fungal communities are primarily dominated by *Candida*.** Principal coordinates analysis (PCoA) of Bray Curtis dissimilarity for n = 26 BAL fungal communities representatively rarefied to 1044 reads/sample demonstrate that **A.** the majority of samples with fungal profiles are dominated by *Candida* (PERMANOVA for dominant genus, R^2^ = 0.673, p < 0.001), and **B.** CD4 count (grouped by quartile with quartiles 2 and 3 combined) is significantly related to fungal composition with *Pneumocystis* dominated patients all possessing low CD4 counts (PERMANOVA, R^2^ = 0.220, p < 0.01). **C.** Taxonomic profiles of fungal microbiota with bacterial dominant family indicated above. *Pneumocystis* dominated samples consistently lacked lower airway 16S bacterial amplification (in white), indicating that patients examined for bacterial pneumonia do not have confounding *Pneumocysis* pneumonia. **D.** Bacterial family dominance may relate to fungal co-colonization (Chi-square, p=0.46). PC = principal coordinate; PERMANOVA = permutational multivariate ANOVA.

**Figure S3.**

**
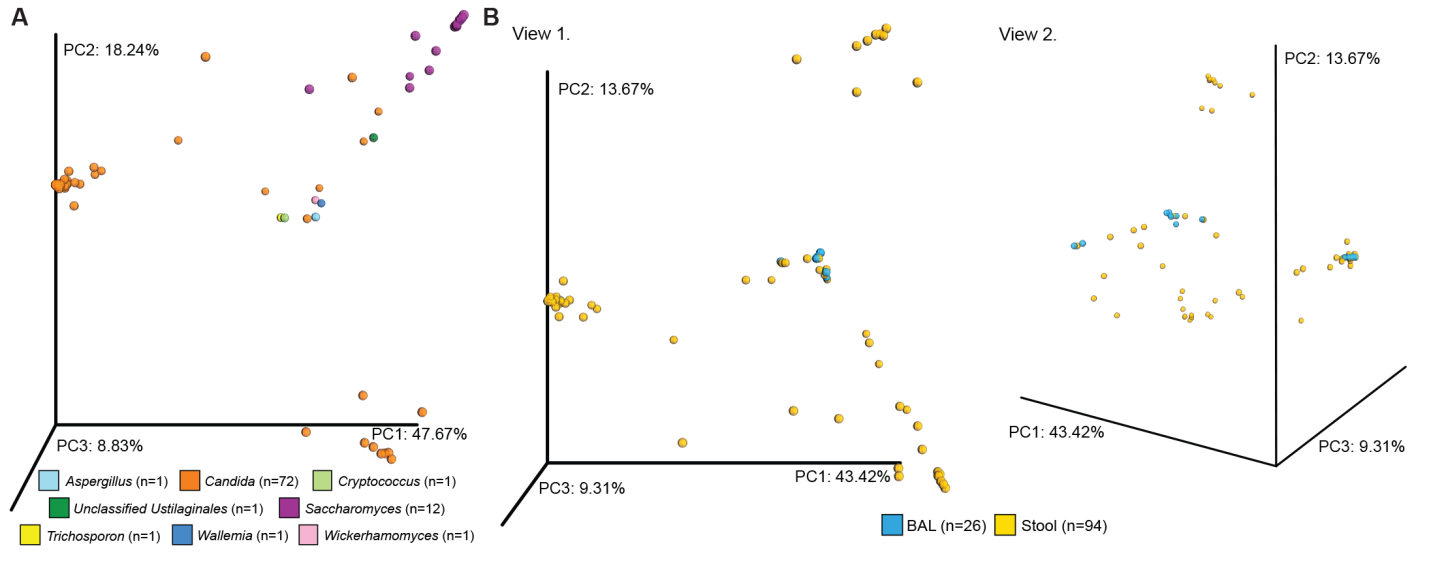
**

**
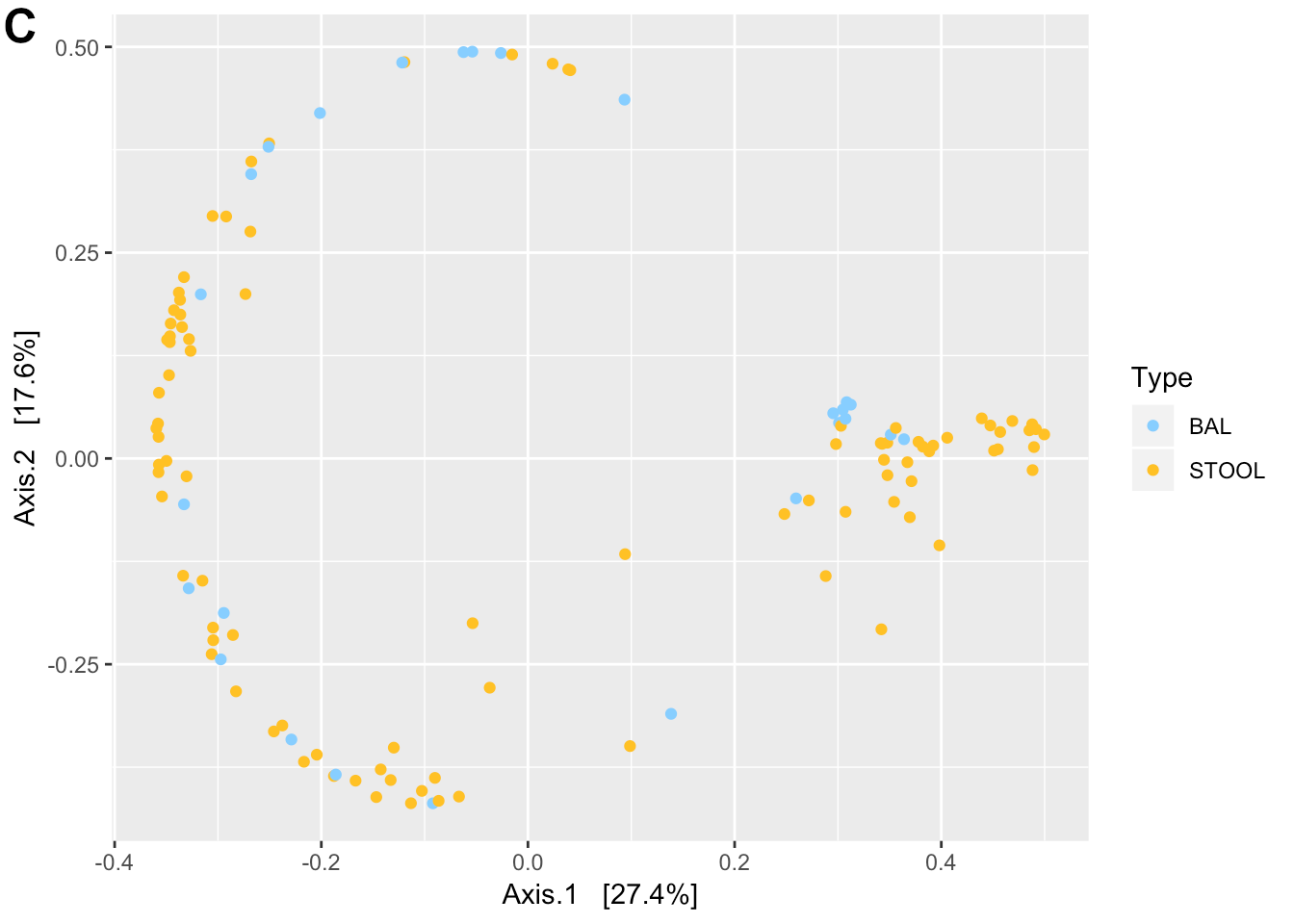
**

**Figure S3. Similar to lower airways, stool of HIV-infected pneumonia patients is consistently dominated by *Candida* and does not show anatomic site distinction from the lower airways. A.** Principal coordinates analysis (PCoA) of Bray Curtis dissimilarity (BC) for n = 90 stool fungal communities representatively rarefied to 2,565 reads/sample demonstrate that the majority of samples with fungal profiles are dominated by *Candida* (PERMANOVA for dominant genus, R^2^ = 0.442, p < 0.001). *Candida* cluster on the left is dominated by OTU 1, cluster on the bottom right by OTU2, and samples in the middle by OTU4. **B.** PCoA of BC for n=26 BAL and n = 94 stool fungal communities representatively rarefied to 1000 reads/sample demonstrate samples are not clustered by anatomic site. Two views are provided to fully display BAL microbiota distribution. **C.** PCoA for all reads from n=26 BAL and n = 94 stool fungal communities, normalized by DESEq2, confirms lack of anatomic site clustering. PC = principal coordinate; PERMANOVA = permutational multivariate ANOVA; BAL = bronchoalveolar lavage.

**Figure S4.**

**
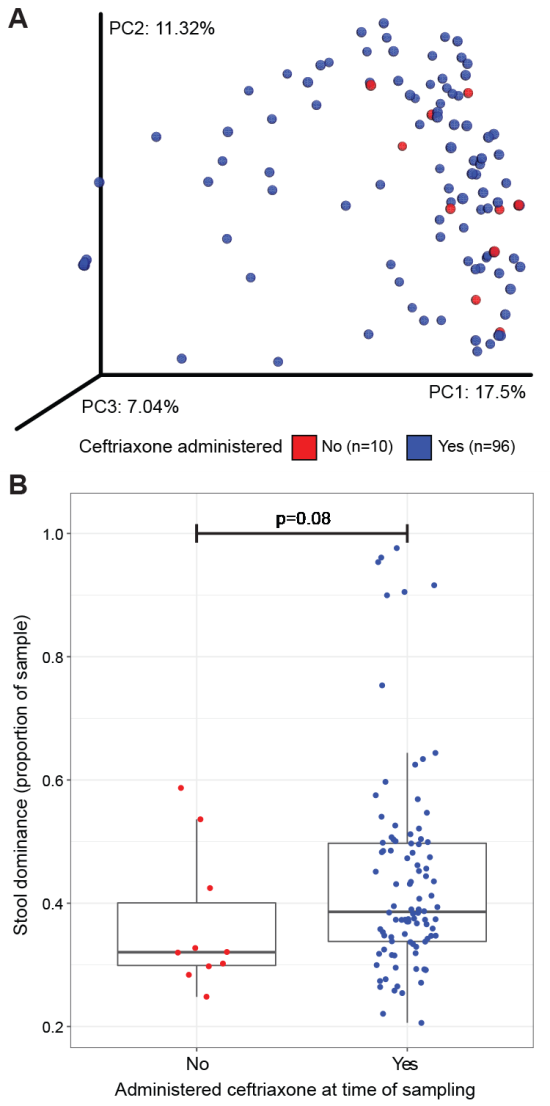
**

**Figure S4. The antibiotic ceftriaxone explains a minor proportion of variance in stool bacterial microbiota.** Principal coordinates analysis (PCoA) of Bray Curtis dissimilarity for n=106 stool samples demonstrates that ceftriaxone administration at time of sample collection significantly explains variation in stool composition (PERMANOVA, R^2^ = 0.019, p = 0.01).

**Figure S5.**

**
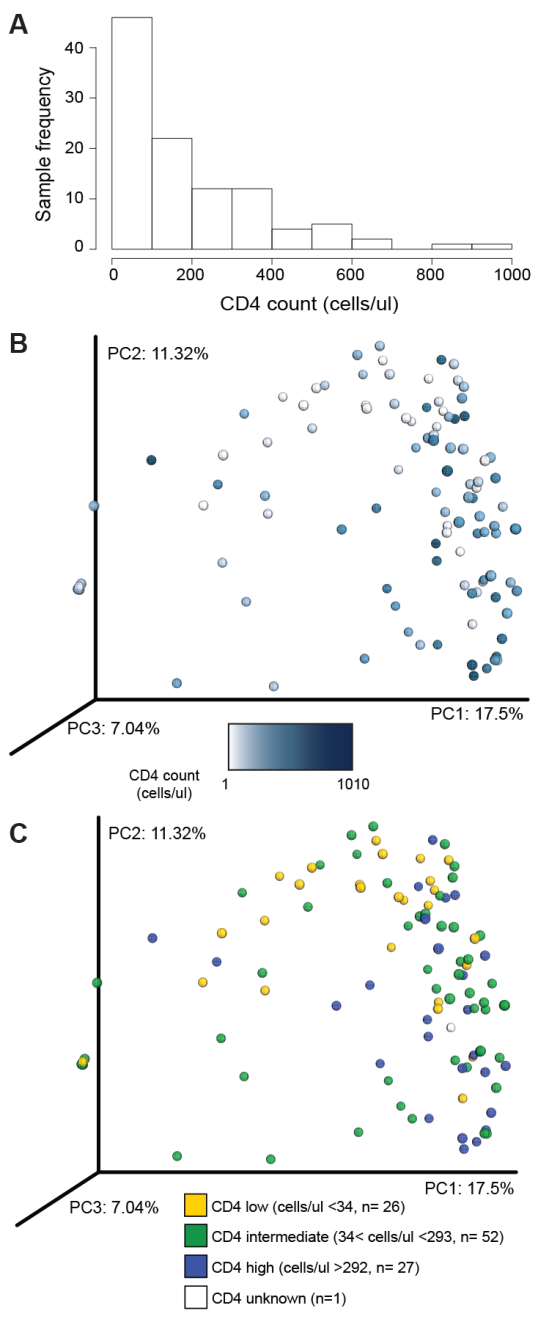
**

**Figure S5. Stool bacterial microbiota is related to circulating CD4 count. A.** Histogram of CD4 count (cells/µl) distribution across patients with stool bacterial profiles. Principal coordinates analysis (PCoA) of Bray Curtis dissimilarity for n = 106 stool samples representatively rarefied to 120,665 reads/sample demonstrates that CD4 count significantly explains variation in stool composition, both as **B.** a continuous variable (cells/μl; PERMANOVA, R^2^ = 0.017, p = 0.025) and **C.** grouped by quartile (second and third combined into intermediate group; PERMANOVA, R^2^ = 0.052, p = 0.002). PC = principal coordinate; PERMANOVA = permutational multivariate ANOVA.

**Figure S6.**

**
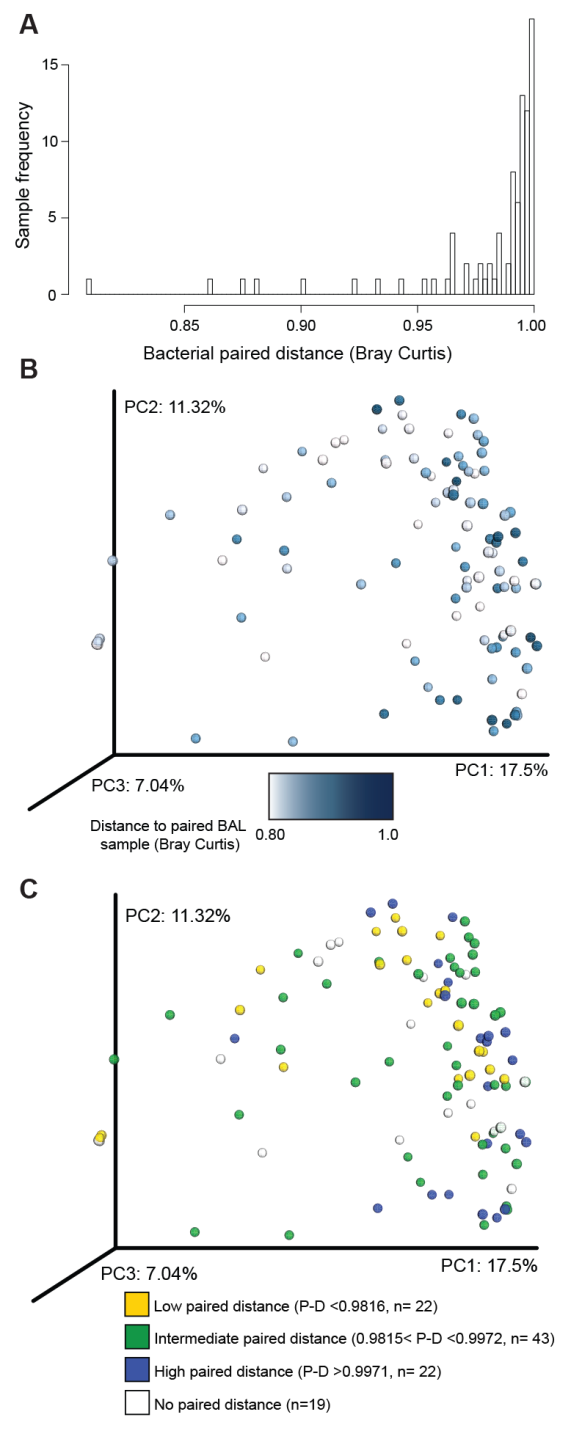
**

**Figure S6. Stool bacterial microbiota composition differs based on similarity to paired BAL sample. A.** Histogram of paired BAL-stool microbiota distance [Bray Curtis (BC)] distribution (outlier distance = 0.8). Principal coordinates analysis (PCoA) of BC for n = 106 stool samples demonstrates that distance to paired BAL sample (BC) significantly explains variation in stool composition, both as **B.** a continuous variable (PERMANOVA, R^2^ = 0.026, p = 0.007) and **C.** grouped by quartile (second and third combined into intermediate group; PERMANOVA, R^2^ = 0.054, p = 0.002). PC = principal coordinate; PERMANOVA = permutational multivariate ANOVA; BAL = bronchoalveolar lavage.**
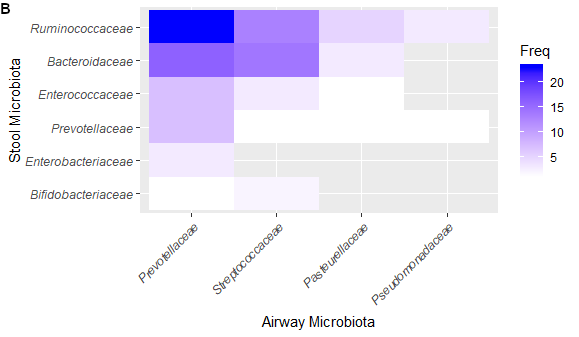

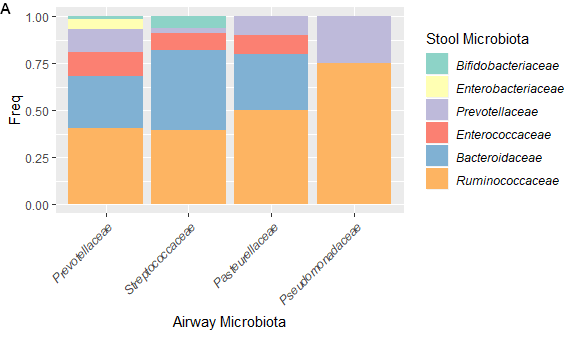
Figure S7.**

**Figure S7.** **BAL and stool dominant bacterial family do not relate to one another within patients. A.** Bar graph plotting frequency of patient dominant airway family stratified by paired stool dominant family. Dominant family is not statistically related between the two anatomic sites. **B.** Heatmap illustrating the frequency of patient airway microbiota that are paired to each stool microbiota. Freq = Frequency.

**Figure S8.**

**
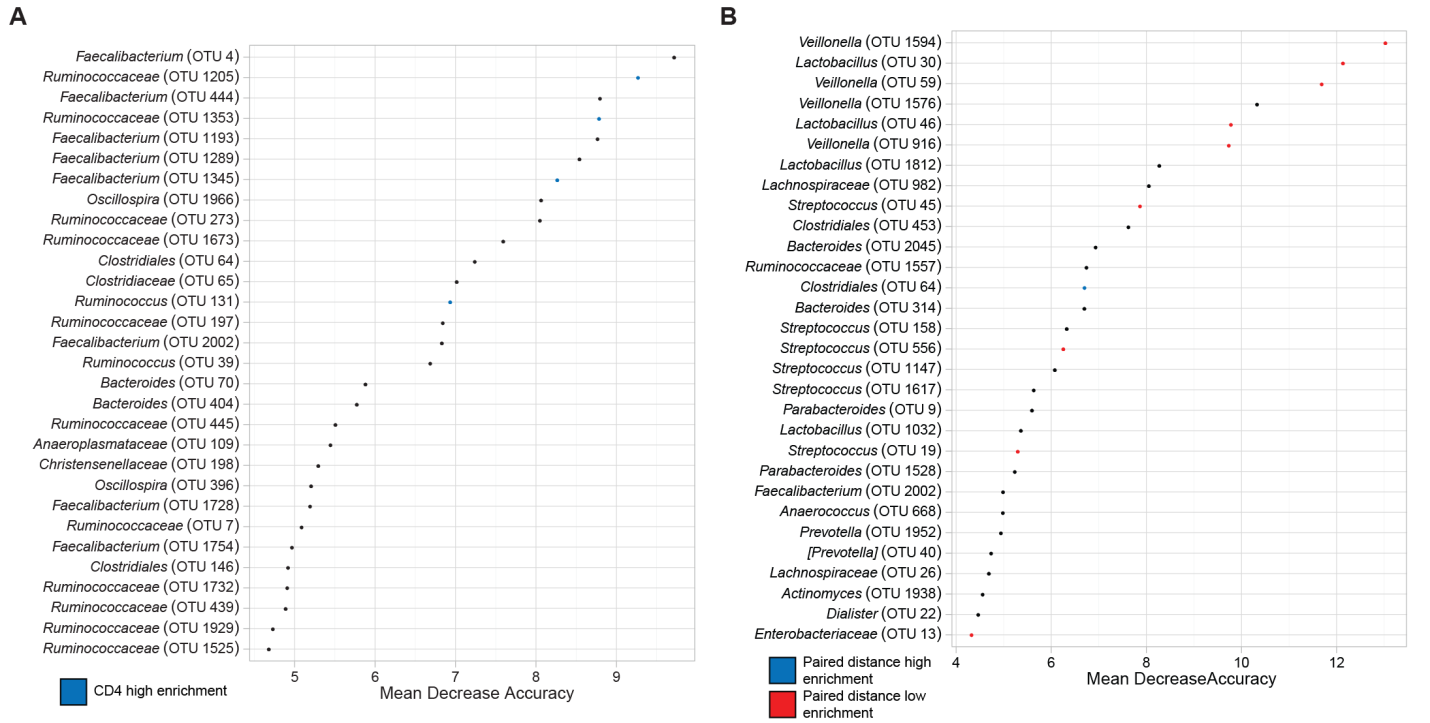
**

**Figure S8. Random Forest confirms CD4 and paired distance associated enrichments.** Random Forest mean decrease accuracy plotted for the top 30 most predictive taxa of CD4 group (quartile 1 versus 4), confirms enrichment of *Faecalibacterium*, *Bacteroides*, and *Ruminococcaceae* in stool bacterial communities of patients with high CD4 counts.

**Figure S9.**

**
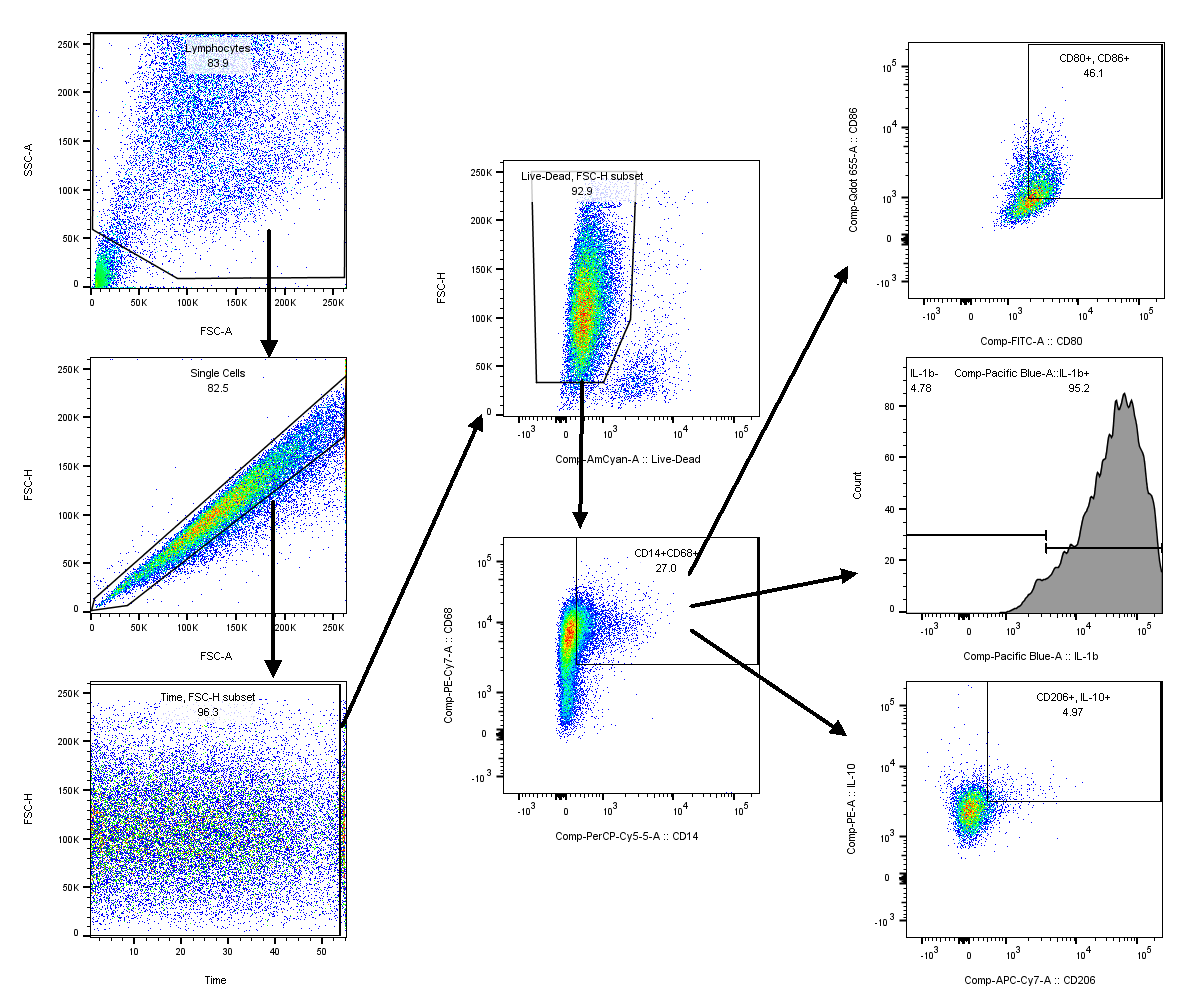
**

**Figure S9. THP-1 flow gating strategy.** THP-1 human monocyte-derived macrophages treated with sterile fecal water were pre-gated to single cell, live macrophages prior to subset gating based on activation marks CD80 and CD86, pro-inflammatory marker IL-1β, and tissue repair markers CD206 and IL-10 (right side, top to bottom respectively).

**Figure S10.**

**
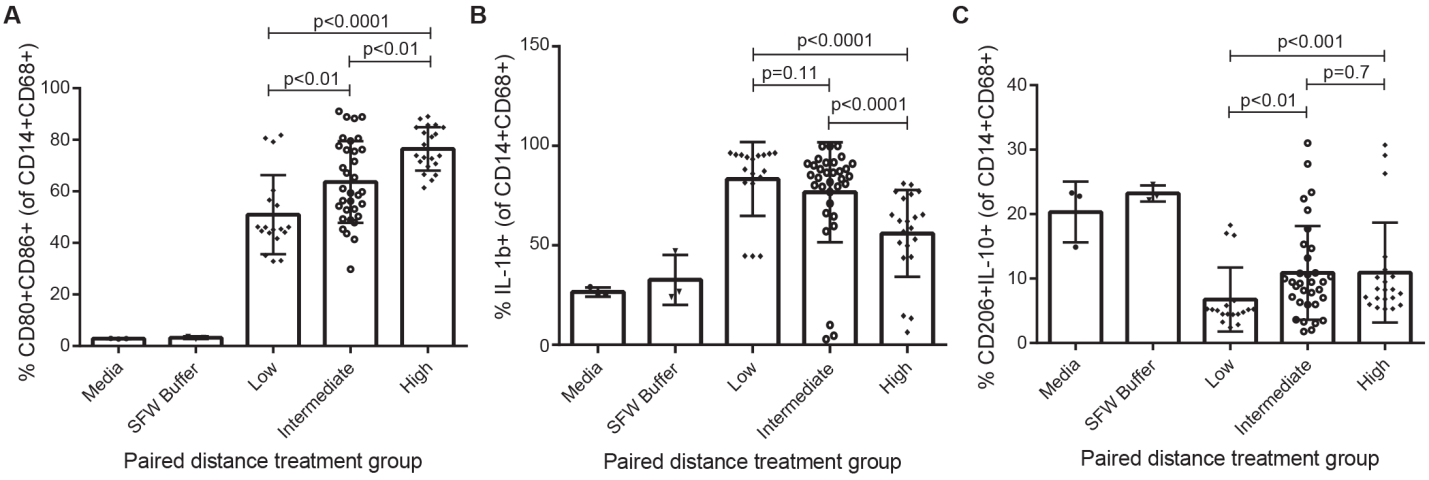
**

**Figure S10. Sterile fecal water from patients with low P-D induce increased activated and pro-inflammatory macrophages and decreased tissue repair macrophage differentiation.** THP-1 human monocyte-derived macrophages treated with low P-D sterile fecal water (SFW) induced increased **A.** activation as measured by CD80+CD86+ cell percentages (KW, p<0.0001; all treatments versus media or buffer p<0.001) and **B.** inflammation as measured by intracellular IL-1β+ cell frequency compared to P-D high SFW (KW, p<0.0001; low or intermediate versus media or buffer p<0.01, high versus media or buffer p<0.08). **C.** Conversely, P-D high SFW induced significantly more CD206+IL-10+ tissue repair macrophages compared to P-D low SFW (KW, p<0.001; low versus media or buffer p<0.05, intermediate or high versus media or buffer p<0.06). Low and high groups are quartiles 1 and 4 respectively, with paired distance quartile 2 and 3 combined into an intermediate group. Data representative of 2 independent experiments; 24 biological replicates with 3 technical replicates each are plotted. Plotted p-values from Mann-Whitney test. KW = Kruskal Wallis; SFW = sterile fecal water.

**Table S1. Clinical and demographic features of the HIV-infected pneumonia patient cohort.**

| **Variable** | **Sample (n)** | **Yes/No^a^** | **Min-Max (Median)** |
| --- | --- | --- | --- |
| Gender | 153 | 77\|76 (F\|M) |  |
| Smoker | 153 | 35\|118 |  |
| CD4 count (cells/µL) | 151 |  | 1-1010 (131) |
| Age | 153 |  | 19-70 (34) |
| Temperature (ºC) | 153 |  | 34.7-41 (36.9) |
| Chest pain | 153 | 97\|56 |  |
| Cough | 153 | 153\|0 |  |
| Wheeze | 153 | 34\|119 |  |
| Previous TB diagnosis | 153 | 19\|134 |  |
| Pulmonary Kaposi's Sarcoma | 146 | 6\|140 |  |
| Pneumocystis prophylaxis | 123 | 101\|22 |  |
| Antiretrovirals at admission | 123 | 64\|59 |  |
| Antibiotics at sample collection | 153 | 153\|0 |  |
| Ceftriaxone at sample collection | 146 | 136\|10 |  |
| ^a^ Unless otherwise noted | | | |

**Table S2. Clinical features across airway microbiota.**

| Variable | *Prevotellaceae* | | *Streptococcaceae* | | *Gammaproteobacteria* | | p-value  (test) |
| --- | --- | --- | --- | --- | --- | --- | --- |
|  | Sample (n) | Yes/No^a^ | Sample (n) | Yes/No^a^ | Sample (n) | Yes/No^a^ |  |
| Mortality at hospital discharge (Survivor\|Deceased) | 60 | 56\|4 (S\|D) | 40 | 40\|0 (S\|D) | 17 | 17\|1 (S\|D) | 0.26  (Chi-squared) |
| Previous TB diagnosis | 60 | 2\|58 | 40 | 3\|37 | 18 | 0\|18 | 0.37  (Chi-squared) |
| Ceftriaxone at sample collection | 60 | 55\|5 | 40 | 36\|4 | 18 | 16\|2 | 0.92  (Chi-squared) |
| CD4 count (cells/ul; median \| range) | 60 | 105 \|  1 – 1010 | 39 | 135 \|  2 - 859 | 18 | 163 \|  1 - 431 | 0.85  (Kruskal-Wallis) |
| CD4 group (High\|Med\|Low) | 60 | 16\|32\|12 (H\|M\|L) | 39 | 7\|18\|14 (H\|M\|L) | 18 | 2\|12\|4 (H\|M\|L) | 0.26  (Chi-squared) |
| Paired distance group (High\|Med\|Low) | 59 | 16\|29\|4 (H\|M\|L) | 37 | 7\|20\|10 (H\|M\|L) | 17 | 3\|11\|3 (H\|M\|L) | 0.62  (Kruskal-Wallis) |
| ^a^ unless otherwise noted |  |  |  |  |  |  |  |

**Table S3. HIV-infected pneumonia patient stool fungal composition is consistently dominated by *Candida* or *Saccharomyces*.**

| **Dominant Genus** | **Sample number (n =)** | **Mean dominance (%)** |
| --- | --- | --- |
| No fungal profile^a^ | 57 | *NA* |
| *Candida* | 72 | 94 |
| *Saccharomyces* | 12 | 82 |
| *Trichosporon* | 1 | 66 |
| *Aspergillus* | 1 | 87 |
| *Cryptococcus* | 1 | 85 |
| *Unclassified Ustilaginales* | 1 | 50 |
| *Wallemia* | 1 | 58 |
| *Wickerhamomyces* | 1 | 24 |
| ^a^Either less amplification than no template controls or did not reach rarefying depth of 2565 reads/sample | | |

| **Table S4. Taxa relatively enriched in the gut microbiota of CD4 low** (white) **versus CD4 high** (gray) **HIV-infected patients** (based on first and fourth quartile respectively; Monaco Study) | | | | | | |
| --- | --- | --- | --- | --- | --- | --- |
| **OTU_ID** | **CD4 High**  **mean** | **CD4 low**  **mean** | **Delta** | **Taxonomy** | **p value** | **q value** |
| OTU_30 | 165 | 3068 | -2903 | *Bacteroidetes; Bacteroidales; S24-7* | 0.003 | 0.028 |
| OTU_32 | 51 | 1532 | -1481 | *Bacteroidetes; Bacteroidales; Bacteroides* | 0.0004 | 0.006 |
| OTU_8 | 0.3 | 1344 | -1344 | *Proteobacteria; Aeromonadales; Ruminobacter* | 5.24E-10 | 2.99E-07 |
| OTU_9 | 245 | 1319 | -1073 | *Firmicutes; Ruminococcaceae* | 0.0002 | 0.003 |
| OTU_62 | 105 | 1097 | -992 | *Bacteroidetes; Parabacteroides* | 0.001 | 0.013 |
| OTU_1112 | 33 | 839 | -805 | *Bacteroidetes; Bacteroides* | 2.71E-05 | 0.0007 |
| OTU_1298 | 8 | 473 | -464 | *Bacteroidetes; Prevotella* | 2.67E-05 | 0.0007 |
| OTU_38 | 13 | 464 | -450 | *Firmicutes; Ruminococcaceae* | 0.001 | 0.011 |
| OTU_34 | 15 | 373 | -357 | *Lentisphaerae; Victivallaceae* | 0.004 | 0.033 |
| OTU_43 | 0.1 | 343 | -343 | *Firmicutes; Veillonellaceae* | 6.83E-09 | 1.30E-06 |
| OTU_1081 | 11 | 224 | -213 | *Firmicutes; Eubacterium* | 0.001 | 0.009 |
| OTU_159 | 0.3 | 210 | -210 | *Tenericutes; RF39* | 1.08E-09 | 3.08E-07 |
| OTU_1195 | 1.6 | 200 | -198 | *Bacteroidetes; Bacteroides* | 9.55E-08 | 6.80E-06 |
| OTU_281 | 8 | 138 | -130 | *Firmicutes; Clostridiales* | 0.0003 | 0.004 |
| OTU_179 | 9 | 135 | -126 | *Firmicutes; Lachnospiraceae* | 0.0016 | 0.016 |
| OTU_239 | 5 | 112 | -107 | *Firmicutes; Clostridiales* | 0.0017 | 0.017 |
| OTU_126 | 6 | 106 | -100 | *Firmicutes; Ruminococcaceae* | 0.0002 | 0.003 |
| OTU_215 | 127 | 0.3 | 126 | *Actinobacteria; Atopobium* | 4.81E-08 | 4.57E-06 |
| OTU_210 | 148 | 2.6 | 146 | *Actinobacteria; Actinomyces* | 2.72E-08 | 3.10E-06 |
| OTU_1508 | 197 | 20 | 177 | *Firmicutes; Veillonella* | 0.002 | 0.019 |
| OTU_1253 | 223 | 17 | 205 | *Firmicutes; Veillonella* | 1.05E-05 | 0.0003 |
| OTU_109 | 238 | 22 | 216 | *Firmicutes; Leuconostocaceae* | 0.001 | 0.0163 |
| OTU_88 | 494 | 31 | 463 | *Firmicutes; Veillonella* | 1.14E-05 | 0.0003 |
| OTU_55 | 626 | 90 | 536 | *Proteobacteria; Haemophilus* | 0.001 | 0.0141 |
| OTU_145 | 695 | 86 | 608 | *Firmicutes; Streptococcus* | 0.000621088 | 0.007867109 |
| OTU_720 | 1205 | 402 | 803 | *Firmicutes; Ruminococcaceae* | 0.002863858 | 0.024364161 |
| OTU_15 | 1599 | 127 | 1471 | *Proteobacteria; Enterobacteriaceae* | 1.25E-06 | 7.12E-05 |
| OTU_26 | 3882 | 28 | 3854 | *Firmicutes; Lactobacillus* | 2.66E-08 | 3.10E-06 |

| **Table S5. Taxa relatively enriched in the gut microbiota of HIV uninfected (white) versus HIV infected (gray) subjects (Monaco Study)** | | | | | | | |
| --- | --- | --- | --- | --- | --- | --- | --- |
| OTU_ID | HIV infected | HIV uninfected | Mean difference | Taxonomy | p value | q value |  |
| OTU_62 | 116 | 607 | -490 | *Bacteroidales; Parabacteroides* | 0.0010 | 0.0352 |  |
| OTU_88 | 25 | 277 | -252 | *Firmicutes; Veillonella* | 3.19E-08 | 6.79E-06 |  |
| OTU_1253 | 27 | 136 | -109 | *Firmicutes; Veillonella* | 0.0001 | 0.0090 |  |
| OTU_650 | 108 | 7.9 | 100 | *Bacteroidetes; Parabacteroides* | 0.0005 | 0.0210 |  |
| OTU_1298 | 218 | 17 | 200 | *Bacteroidetes; Prevotella* | 0.0001 | 0.0079 |  |
| OTU_48 | 415 | 146. | 268 | *Firmicutes; Lachnospiraceae* | 0.0005 | 0.0202 |  |
| OTU_1496 | 283 | 12.6 | 271 | *Bacteroidetes; Prevotella* | 3.30E-06 | 0.0003 |  |
| OTU_17 | 569 | 206 | 362 | *Firmicutes; Ruminococcaceae* | 0.0002 | 0.0126 |  |
| OTU_1258 | 663 | 177 | 486 | *Firmicutes; Roseburia* | 0.0005 | 0.0202 |  |
| OTU_35 | 708 | 80 | 627 | *Firmicutes; Lachnospiraceae* | 4.92E-09 | 3.15E-06 |  |
